# Exploration and functionalization of M1-macrophage extracellular vesicles for effective accumulation in glioblastoma and strong synergistic therapeutic effects

**DOI:** 10.1038/s41392-022-00894-3

**Published:** 2022-03-16

**Authors:** Xiaojun Wang, Hui Ding, Zongyang Li, Yaonan Peng, Hui Tan, Changlong Wang, Guodong Huang, Weiping Li, Guanghui Ma, Wei Wei

**Affiliations:** 1 Shenzhen Key Laboratory of Neurosurgery, Department of Neurosurgery, Shenzhen Second People’s Hospital, Shenzhen University 1st Affiliated Hospital, Guangdong, 518035 P. R. China; 2 State Key Laboratory of Biochemical Engineering, Institute of Process Engineering, Chinese Academy of Sciences, Beijing, 100190 P. R. China; 3 Department of Ophthalmology, Beijing Chaoyang Hospital, Capital Medical University, Beijing, 100020 P. R. China; 4 Department of Otolaryngology and Institute of Translational Medicine, Shenzhen Second People’s Hospital, the First Affiliated Hospital of Shenzhen University Health Science Center, Shenzhen, Guangdong, 518110 P. R. China; 5 Department of Neurosurgery, Jinling Hospital, Jiangsu, Nanjing, 210000 P. R. China; 6 School of Chemical Engineering, University of Chinese Academy of Sciences, Beijing, 100049 P. R. China

**Keywords:** Cancer therapy, Drug delivery

## Abstract

Glioblastoma multiforme (GBM) is a highly aggressive brain tumor with an extremely low survival rate. New and effective approaches for treatment are therefore urgently needed. Here, we successfully developed M1-like macrophage-derived extracellular vesicles (M1EVs) that overcome multiple challenges via guidance from two macrophage-related observations in clinical specimens from GBM patients: enrichment of M2 macrophages in GBM; and origination of a majority of infiltrating macrophage from peripheral blood. To maximize the synergistic effect, we further functionalized the membranes of M1EVs with two hydrophobic agents (the chemical excitation source CPPO (C) and the photosensitizer Ce6 (C)) and loaded the hydrophilic hypoxia-activated prodrug AQ4N (A) into the inner core of the M1EVs. After intravenous injection, the inherent nature of M1-derived extracellular vesicles CCA-M1EVs allowed for blood-brain barrier penetration, and modulated the immunosuppressive tumor microenvironment via M2-to-M1 polarization, which increased hydrogen peroxide (H_2_O_2_) levels. Furthermore, the reaction between H_2_O_2_ and CPPO produced chemical energy, which could be used for Ce6 activation to generate large amounts of reactive oxygen species to achieve chemiexcited photodynamic therapy (CDT). As this reaction consumed oxygen, the aggravation of tumor hypoxia also led to the conversion of non-toxic AQ4N into toxic AQ4 for chemotherapy. Therefore, CCA-M1EVs achieved synergistic immunomodulation, CDT, and hypoxia-activated chemotherapy in GBM to exert a potent therapeutic effect. Finally, we demonstrated the excellent effect of CCA-M1EVs against GBM in cell-derived xenograft and patient-derived xenograft models, underscoring the strong potential of our highly flexible M1EVs system to support multi-modal therapies for difficult-to-treat GBM.

## Introduction

Glioblastoma multiforme (GBM), the most aggressive type of brain tumor, is characterized by a poor prognosis, an extremely high mortality rate, and a high tendency for recurrence.^1–3^ Despite advances in surgery, radiation, and chemotherapy, the median survival time of GBM is only 15–16 months, and the 5-year overall survival rate is less than 5%.^4–6^ Thus, there is an urgent need to develop new effective therapeutic strategies for treating GBM.^7^

Treatments comprising a single therapeutic modality (mono-therapy) are often insufficient to effectively kill tumor cells. Accordingly, there is a great deal of ongoing research on the development, testing, and application of multi-modal antitumor treatments, including many promising studies on immunotherapy agents for combination therapies for the treatment of diverse tumors.^8–11^ However, some treatment strategies do not exhibit good synergy to achieve the best therapeutic efficiency. Considering multi-modal treatments for GBM, there are at least two other major, well-understood physiological processes that limit the efficacy of treatments against GBM.^12^ The most prominent limitation is the blood-brain barrier (BBB), which separates brain tissue from circulating blood.^13^ Over 98% of therapeutic agents are prevented from reaching the brain, precluding their application for the treatment of GBM.^14,15^ Another challenge is that some strategies used in multi-modal approaches are not suitable for the treatment of GBM.^16^ For example, the therapeutic effects of photothermal therapy (PTT) and photodynamic therapy (PDT) for GBM are restricted by the depth of penetration of laser irradiation in the brain.^17,18^

**Scheme 1:**
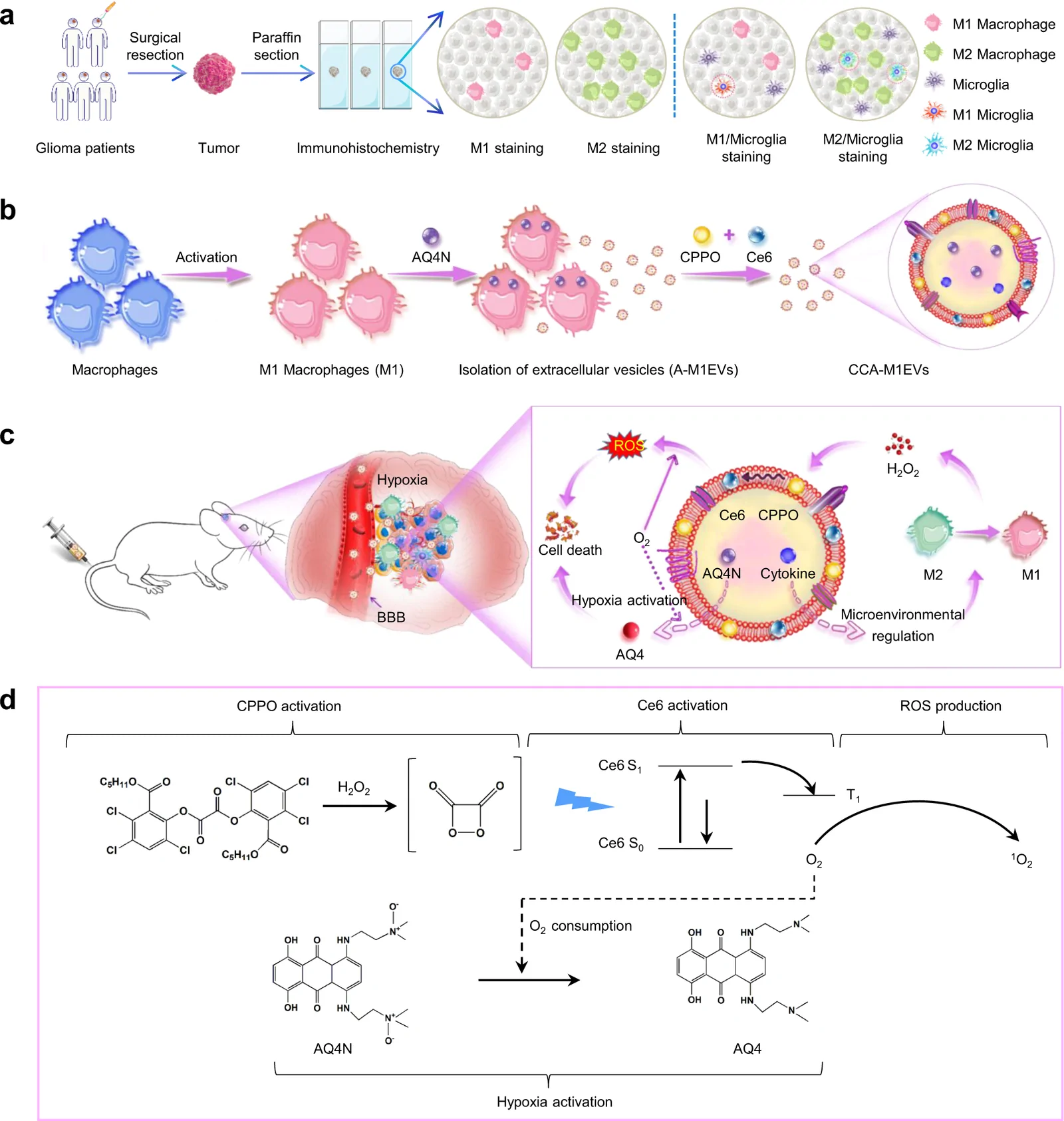
Scheme of clinical glioma analysis, exosomal formulation construction, and tumor inhibition mechanism. **a**. Tumor-associated macrophages (TAMs) phenotype analysis of tumor samples from glioma patients. **b**. Schematic illustration of the formation of CCA-M1EVs. **c**. Accumulation in gliomas and synergism of immunomodulation, chemiexcited photodynamic therapy, and hypoxia-triggered chemotherapy of CCA-M1EVs in a murine model of gliomas. **d**. Corresponding illustration of chemical reactions induced by CCA-M1EVs

By analyzing clinical samples (Scheme 1a), we observed an increase in the pool of M2-like macrophages in GBM, which was confirmed by the CD163-positive phenotype. In contrast, fewer M1-like macrophages were found in GBM samples, as confirmed by an iNOS-positive phenotype. In addition, we found that infiltrated macrophages were mainly peripheral blood-derived macrophages, as confirmed by a TMEM119-negative phenotype. Based on this observation, we considered using M1-like macrophages to modulate the tumor microenvironment (TME). However, extensive studies have demonstrated that antitumor M1-like macrophages might typically switch to an immunosuppressive tumor-associated macrophage (TAM) phenotype when these cells infiltrate the tumor site.^19,20^ We therefore envisioned using M1 macrophage-derived extracellular vesicles (M1EVs) as an alternative strategy to develop therapeutics against GBM. In theory, an M1EVs strategy would offer the well-demonstrated benefits of EVs from endogenous cells compared to many other types of nanomedicine delivery platforms, including their high biocompatibility and unique capacity to retain the highly complex biological functions of mother cells (such as chemotaxis and immunomodulatory regulation).^21–27^ These benefits suggest that M1EVs are much more reliable in modulating M2-to-M1 polarization. In addition, similar to lipid vesicles, EVs can be engineered to deliver different drug molecules due to their hollow and bilayer structure.^28^

Herein, we report the development of M1EVs as a drug delivery system for GBM treatment. After initial analysis of their accumulation at GBM tumor sites and M2-to-M1 polarization capacities, M1-like macrophages were incubated with the inactivated chemotherapy agent banoxantrone (AQ4N, A) followed by release and isolation of M1EVs carrying AQ4N in the inner core (A-M1EVs) that was loaded using a method as reported previously.^29–32^ These drug-loaded EVs were subsequently elaborated with hydrophobic bis(2,4,5-trichloro-6-carbopentoxyphenyl) oxalate (CPPO, C) and chlorin e6 (Ce6, C) in their membranes to obtain CCA-M1EVs (Scheme 1b). After accumulating in GBM tumors, CCA-M1EVs induced M2-to-M1 polarization for TME immunomodulation, leading to increased H_2_O_2_ levels. Furthermore, chemical energy was produced by the reaction between H_2_O_2_ and CPPO, and this energy could be used for Ce6 activation to generate large amounts of reactive oxygen species (ROS) to achieve chemiexcited photodynamic therapy (CDT).^33^ As this reaction consumed oxygen, aggravation of tumor hypoxia also led to the conversion of non-toxic AQ4N to toxic AQ4 for chemotherapy (Scheme 1c, d). Subsequently, we tested the synergism of immunomodulation, CDT, hypoxia-activated chemotherapy, and overall anti-GBM tumor effect of the CCA-M1EVs in GBM cells, cell-derived xenograft (CDX) tumors, and patient-derived xenograft (PDX) tumors.

## Results

### High expression of M2 macrophages and origination of most infiltrated macrophage from peripheral blood in glioma patients and glioma-bearing mice

**Fig. 1:**
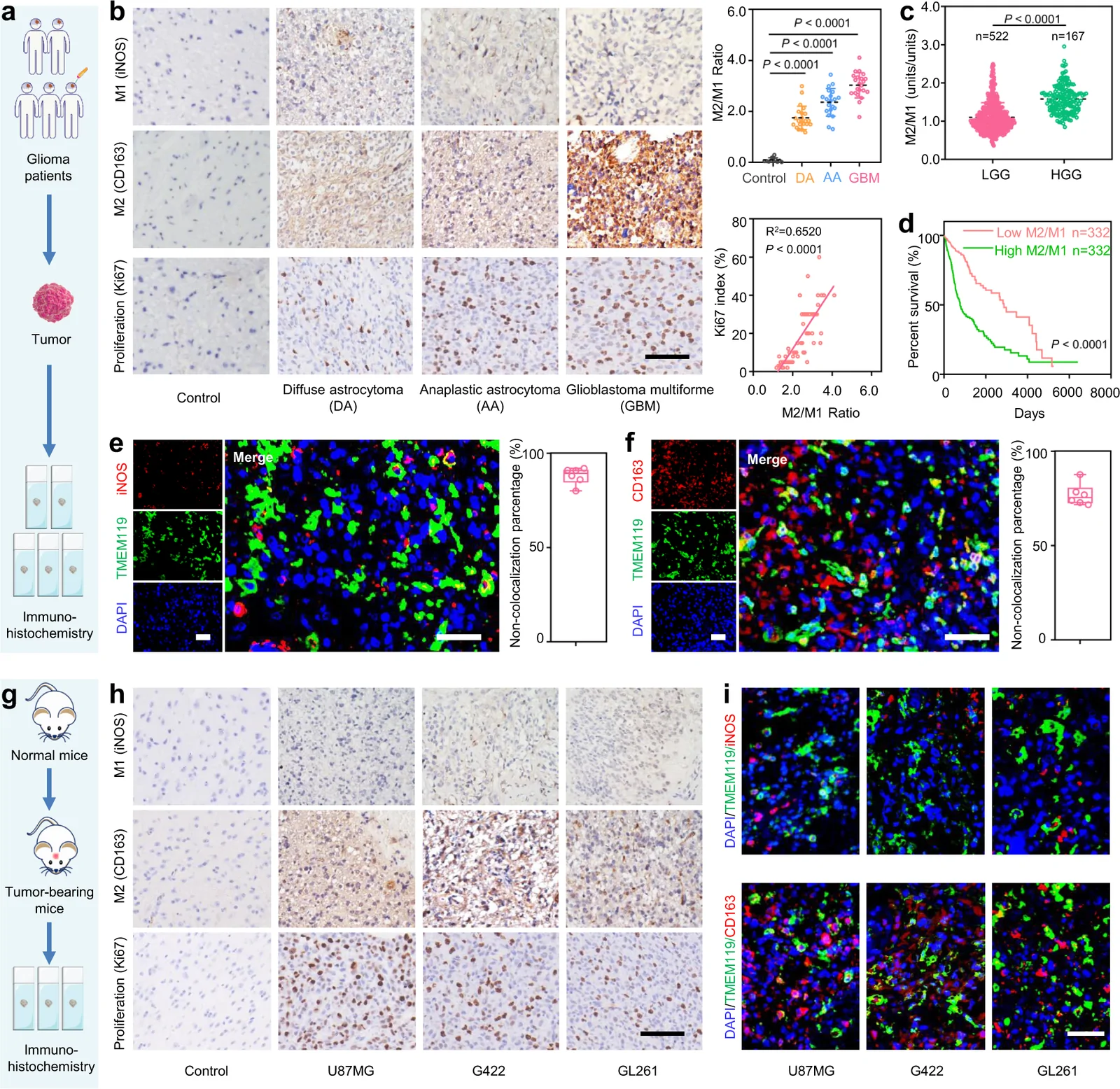
Analysis of diverse gliomas obtained from patients and mice revealed distinct TAMs phenotypes and their origination. **a**. Schematic illustration of TAMs phenotype analysis from tumor samples of glioma patients. **b**. M1 macrophage (iNOS), M2 macrophage (CD163), and proliferation (Ki67) immunostaining of histological sections of tumor-adjacent tissues as control and in both low-grade gliomas (LGG: diffuse astrocytoma, *n* = 22) and high-grade gliomas (HGG: anaplastic astrocytoma, *n* = 20; glioblastoma multiforme, *n* = 22) resected from glioma patients. Quantitative analysis of the corresponding M2/M1 ratios was shown on the right side. The proliferation-related Ki67 marker index was positively correlated with the M2/M1 ratio. All images have the same scale of 50 μm. **c**. M2/M1 ratio analysis of 167 HGG and 522 LGG cases acquired from The Cancer Genome Atlas (TCGA) database. Each dot represented a single individual. **d**. Survival curves of glioma patients from TCGA database. The OncoLnc tool was used to explore the survival correlations for M2/M1 ratio data. **e**. Immunostaining of histological sections (left) and quantitative analysis (right) of noncolocalization percentage of microglia (TMEM119, green) and M1 macrophage (iNOS, red) of human glioma tissue. All images have the same scale of 50 μm. Nuclei: DAPI, blue (*n* = 6). **f**. Immunostaining of histological sections (left) and quantitative analysis (right) of noncolocalization percentage of microglia (TMEM119, green) and M2 macrophage (CD163, red) of human glioma tissue. All images have the same scale of 50 μm. Nuclei: DAPI, blue (*n* = 6). **g**. Schematic illustration of TAM phenotype analysis from tumor samples of U87MG (human glioblastoma cells) /G422 (mouse glioblastoma cells) /GL261 (mouse glioma cells)-cell-derived xenograft tumor-bearing mice. **h**. M1 macrophage (iNOS), M2 macrophage (CD163), and proliferation (Ki67) immunostaining of histological sections of normal tissue, U87MG, G422, and GL261-bearing tissue in mice. All images have the same scale of 50 μm. **i**. Microglia (TMEM119, green) and M1 macrophage (iNOS, red) immunostaining of histological sections of U87MG, G422, and GL261-bearing tissue (Top). Microglia and M2 macrophage (CD163, red) immunostaining of histological sections of U87MG, G422, and GL261-bearing tissue (bottom). All images have the same scale of 50 μm. Nuclei: DAPI, blue. Data in **b**, **e**, and **f** are presented as the mean ± S.D. Statistical significance was calculated *via* one-way ANOVA with a Tukey post hoc test (**b**) or unpaired two-tailed Student’s *t*-test (**c**) and survival analysis was calculated by two-sided Log-rank Mantel-Cox tests (**d**)

Given reports that the infiltration of activated immune cells into the glioma microenvironment can notably restrict tumor growth and increase patient survival time,^34–36^ we collected tumor samples from 64 cases of human glioma patients, which was classified by WHO classification, including low-grade glioma (LGG: diffuse astrocytoma, DA) and high-grade glioma (HGG: anaplastic astrocytoma, AA; GBM). Immunohistochemistry (IHC) was used to quantify TAMs (Fig. 1a). Subsequently, we evaluated the M1 (iNOS) amount, M2 (CD163) amount, and the rate of proliferation (Ki67) in the samples. The M2/M1 ratio was considerably higher in the GBM group than in the other groups. There was also a strong positive correlation between the M2/M1 ratio and glioma proliferation (Fig. 1b). In addition, the M2/M1 ratio was investigated in glioma cases from The Cancer Genome Atlas (TCGA), including 167 cases of HGG and 522 cases of LGG, and was significantly higher (p < 0.0001) in the HGG group than in the LGG group (Fig. 1c). The results shown here are in whole based upon data generated by the TCGA Research Network: http://www.cancer.gov/tcga. A Kaplan-Meier survival curve investigation of M2/M1 ratio with mortality indicated that glioma patients with lower M2/M1 ratios had better survival outcomes (Fig. 1d). This analysis of TGCA mRNA expression data confirmed the findings from our IHC analysis of clinically resected glioma tissues: a high M2/M1 ratio is apparently an adverse prognostic factor for glioma.

After confirming that a high M2/M1 ratio promoted tumor proliferation, we wanted to identify the source of tumor-infiltrating TAMs in resected clinical glioma tissues. There may be two possible sources: brain-resident microglia or macrophages that have differentiated from peripheral blood-derived monocytes and have penetrated the BBB.^37^ To investigate the above hypothesis, we co-stained M1-like (iNOS) macrophages or M2-like (CD163) macrophages with microglia (TMEM119) in resected samples. The signals of M1-like macrophages (95.4%) and M2-like macrophages (72.7%) did not co-localize with those of microglia, suggesting high infiltration of macrophages might be derived from peripheral blood (Fig. 1e, f).

To confirm the above clinical findings in mice, we employed a mouse glioma model and used IHC to detect TAMs (Fig. 1g). In agreement with the observation in patients, we found that the M2/M1 ratio was high in U87MG (human glioblastoma cells), G422 (mouse glioblastoma cells), and GL261 (mouse glioma cells) tumor model (Fig. 1h and Supplementary Fig. S1a). We further analyzed the source of the tumor-infiltrating TAMs. The results revealed that the majority of TAMs residing in the glioma TME did not co-localize with microglia, which again demonstrated that most infiltrating macrophages were derived from peripheral blood (Fig.1i and Supplementary Fig. S1b). These clinical findings and confirmatory experimental results suggested that it might be possible to deliver M1-like macrophages into GBM tissues and further modulate the immunosuppressive TME to promote antitumor effects. However, it is well-known that M1-like macrophages frequently turn “traitor” to M2-like phenotypes after these cells have infiltrated a tumor site.^38^ We therefore envisioned using EVs derived from M1-like macrophages as a strategy to facilitate both targeted drug delivery to tumors through the BBB and immunomodulatory anti-glioma treatment.

### In vivo targeting and immunomodulation performance

**Fig. 2:**
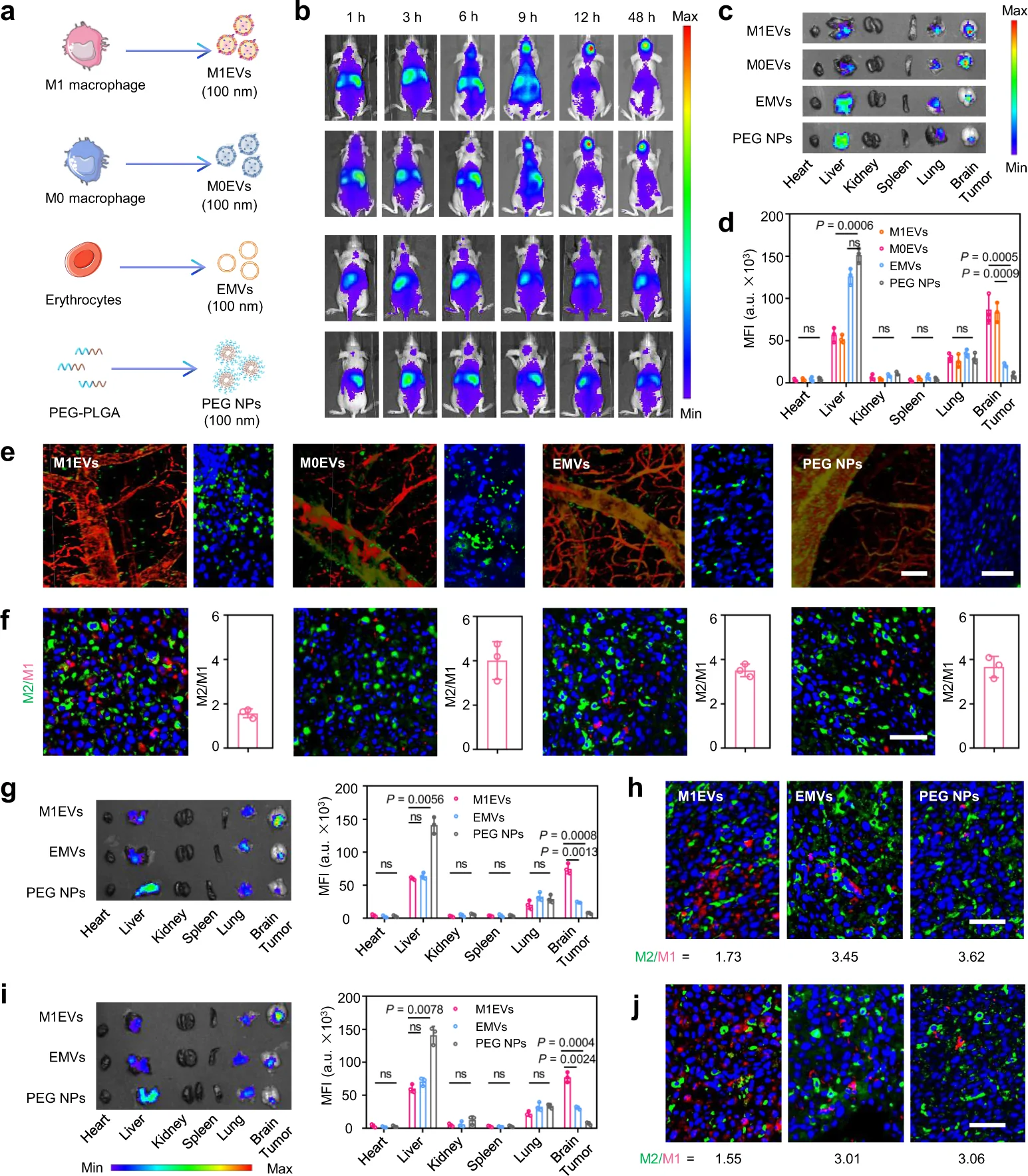
Macrophage-derived EVs could penetrate the blood-brain barrier (BBB) and efficiently modulated the tumor microenvironment (TME). **a**. Schematic illustration of the fabrication process for M1EVs (derived from M1 macrophages), M0EVs (derived from M0 macrophages), EMVs (derived from erythrocytes), and PEG NPs (derived from PEG-PLGA materials). **b**. Representative fluorescence images of U87MG-bearing mice after intravenous (*i.v*.) injection with M1EVs, M0EVs, EMVs, and PEG NPs (all labeled with DiR) at different time points. **c**. Ex vivo images of the major organs dissected from mice in different groups at 48 h after *i.v*. injection. **d**. Quantitative analysis of corresponding fluorescence signals in panel (**c**). **e**. In vivo time-lapse two-photon images of the diffusion of M1EVs, M0EVs, EMVs, and PEG NPs acrossed the brain microvascular endothelial cells at 48 h after *i.v*. injection (left). Tetramethylrhodamine isothiocyanate-Dextran was used to label blood vessels (red). M1EVs, M0EVs, and EMVs labeled with DiO (green); PEG NPs labeled with FITC (green), and corresponding formulation distributions in tumor tissue (right). All images have the same scale of 50 μm. **f**. Immunofluorescence images of histological sections of M2 and M1 macrophages (left), and quantitative analysis of M2/M1 ratios (right) at 48 h after *i.v*. injection. All images have the same scale of 50 μm. iNOS (red, M1 marker), CD163 (green, M2 marker) (*n* = 3). **g**. Ex vivo images of the major organs dissected from G422 bearing mice in different groups (mouse source) at 48 h after *i.v*. injection (left), and corresponding analysis of ex vivo fluorescence signals of the major organs dissected from mice in different groups (right). **h**. Immunofluorescence images of histological sections (G422-bearing mice) of M2 and M1 macrophages (right). Scale bar: 50 μm, iNOS (red, M1 marker), CD163 (green, M2 marker) (*n* = 3). **i**. Ex vivo images of the major organs dissected from GL261 bearing-mice in different groups at 48 h after *i.v*. injection (left), and corresponding analysis of ex vivo fluorescence signals of the major organs dissected from mice in different groups (right). **j**. Immunofluorescence images of histological sections (GL261-bearing mice) of M2 and M1 macrophages (right). Scale bar: 50 μm, iNOS (red, M1 marker), CD163 (green, M2 marker) (*n* = 3). Data in **d**, **f**, **g**, and **i** are presented as the mean ± S.D. Statistical significance was calculated, compared with the M1EVs group, by one-way ANOVA with a Kruskal-Wallis test (**d**, **g**, **i**). ns, not significant

In our initial experimental explorations of this macrophage-based EVs strategy, we prepared a set of four different particles that were all approximately 100 nm in size (all labeled with DiR to facilitate in vivo imaging): vesicles prepared from M1-like macrophages (M1EVs); vesicles prepared from resting macrophages (M0EVs); vesicles prepared from cultured erythrocytes (EMVs); and PEG nanoparticles prepared from self-assembly by PEG-PLGA (PEG NPs) (Fig. 2a). We injected the materials into nude mice bearing orthotopic luciferase-tagged U87MG cells and monitored the biodistribution of different formulations. Due to the chemokine receptors on the MEVs and the corresponding chemokines produced by glioma (Supplementary Fig. S2), the tumor accumulation of M0EVs and M1EVs was more pronounced than that of the EMVs and PEG NPs (Fig. 2b). Twelve hours after injection of M1EVs and M0EVs, the fluorescence intensity (FI) reached a maximum and maintained the high level of fluorescence for up to 48 h (Supplementary Fig. S3). These findings were also confirmed and extended by ex vivo imaging of major organs dissected from mice (Fig. 2c). In particular, the concentration of M1EVs in brain tumor was approximately four times and nine times higher than that of EMVs and PEG NPs, respectively (Fig. 2d).

Subsequently, we conducted two-photon intravital live imaging of GBM model mice through a cranial window. This technique enabled observation of BBB penetration by DiO- or FITC-labeled materials, and we found that many M1EVs and M0EVs infiltrated into the tumor, while EMVs and PEG NPs exhibited less brain accumulation (Fig. 2e and Supplementary Fig. S4a). To achieve a higher resolution, all of the mice were sacrificed, and their brains were collected for frozen-section immunofluorescence (IF) staining. Compared with the sparse signal of EMVs and PEG NPs, the substantial signal of M1EVs occurred throughout the tumor tissue (Supplementary Fig. S4b), which again demonstrated that M1EVs can be utilized for GBM-targeted delivery. To further study the immunomodulation function of M1EVs, tumor slices were stained for M1 and M2 macrophage markers, and this analysis revealed that M1EVs could be successfully immunomodulate M2-to-M1 polarization. The M2/M1 ratio in the M1EVs group (~1.64) was lower compared with M0EVs (~3.82), EMVs (~3.50), and PEG NPs (~3.75) groups (Fig. 2f). Taken together, these findings suggest that M1EVs were far better than the other particles that we examined here in terms of GBM tissue targeting and immunomodulation.

To confirm this superior targeting performance, we further constructed mice bearing orthotropic luciferase-tagged G422/GL261 tumors and comparatively evaluated the biodistribution of three types of particles: extracellular vesicles from M1-like macrophages (M1EVs, mouse source); EMVs; and PEG NPs. Similarly, in vivo and ex vivo imaging showed that the tumor accumulation of M1EVs was more pronounced than that of EMVs and PEG NPs (Supplementary Fig. S5). Quantitatively, the concentration of M1EVs in brain tumor was about four times and ten times higher than that of EMVs and PEG NPs, respectively (Fig. 2g, i). In addition, M1EVs could successfully immunomodulate M2-to-M1 polarization, verified by the higher M2/M1 ratio (Fig. 2h, j). Therefore, our M1EVs platform achieves specific and highly efficient delivery in U87MG-, G422-, and GL261-bearing glioma models.

### Construction, characterizations, evaluations of the penetration capacity, and in vitro synergistic anti-tumor efficacy of CCA-M1EVs

**Fig. 3:**
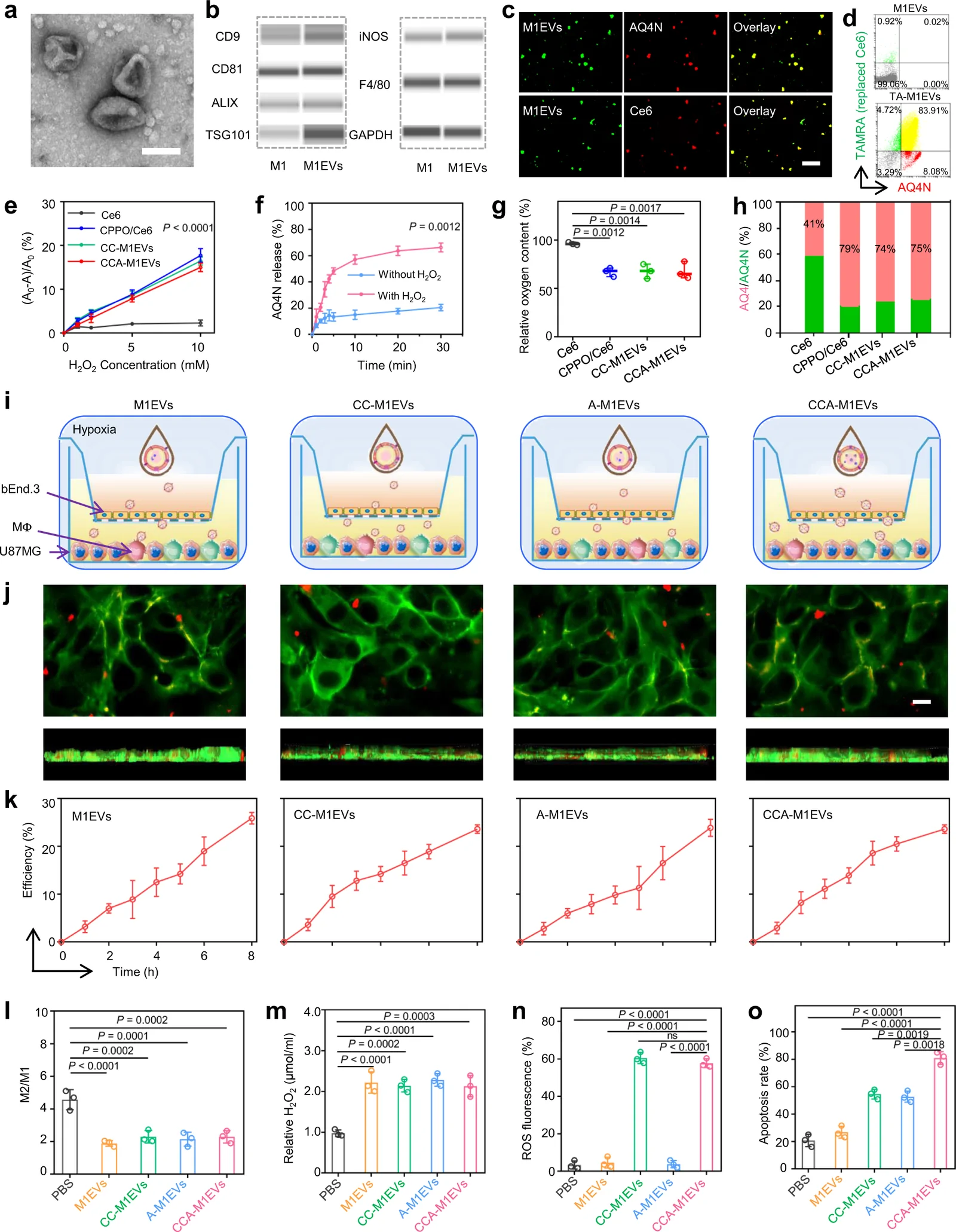
Characterizations of M1EVs based formulations and evaluations of the penetration capacity and synergistic anti-tumor efficacy in vitro. **a**. TEM image of M1EVs. Scale bar: 100 nm. **b**. ProteinSimple^®^ capillary immunoassay (Wes) analysis of CD9, CD81, ALIX, TSG101, iNOS, F4/80, and GAPDH in M1 macrophages and M1EVs. **c**. Confocal laser scanning microscopy (CLSM) images of AQ4N-M1EVs (Top, green: M1EVs; red: AQ4N) and Ce6-M1EVs (bottom, green: M1EVs; red: Ce6). All images have the same scale of 1 μm. **d**. Representative flow cytometry analysis images of M1EVs (top) and TA-M1EVs (M1EVs containing AQ4N and TRMRA in place of Ce6 due to the overlayed spectrum with AQ4N) (bottom). **e**. Production of ROS with Ce6, CPPO/Ce6, CC-M1EVs, and CCA-M1EVs in buffers with different H_2_O_2_ concentrations, where A_0_ and A were the absorbance of ABDA at 399 nm before and after H_2_O_2_ addition (*n* = 3). **f**. Cumulative AQ4N release profiles of CCA-M1EVs before and after H_2_O_2_ treatment in PBS buffer (*n* = 3). **g**. Consumption of oxygen with different formulations after H_2_O_2_ treatment in PBS buffer (*n* = 3). **h**. Quantification of the AQ4/AQ4N ratio after different treatments based on high-performance liquid chromatography (HPLC) analysis. **i**. Illustration of in vitro BBB and TME model. The Transwell^TM^ co-culture system containing bEnd.3 cells in the upper chamber and a combination of U87MG glioma cells and macrophages in the bottom chamber under hypoxic condition. **j**. CLSM images of bEnd.3 cells with different treatments. Scale bar: 5 μm. (green: ZO-1, red: EVs). **k**. Accumulative penetration efficiency of M1EVs, CC-M1EVs, A-M1EVs, and CCA-M1EVs labeled with DiD through a monolayer bEnd.3 layer at different time points (*n* = 3). **l**. Flow cytometry analysis of the M2/M1 ratio in the lower chamber after incubation with different EV designs (*n* = 3). **m**. Production of H_2_O_2_ with different treatments in the lower chamber (Amplex Red Hydrogen Peroxide Assay Kit) (*n* = 3). **n**. Assessment of intracellular ROS (labeled by DCFH-DA) of U87MG cells in the lower chamber (*n* = 3). **o**. Flow cytometry analysis of the cell-death-inducing effect of different formulations on U87MG cells in the lower chamber (Annexin V and PI in the dead cell apoptosis kit) (*n* = 3). Statistical significance was calculated *via* one-way ANOVA with a Kruskal-Wallis test (**e**, **g**, **l**, **m**, **n**, and **o**) or unpaired two-tailed Student’s *t*-test (**f**). ns, not significant

The above-mentioned results prompted us to build a delivery system based on M1EVs. M1-like macrophages were incubated with AQ4N to obtain M1EVs loaded with AQ4N in the core (A-M1EVs). Subsequently, CPPO and Ce6 were loaded on the lipid membrane though incubation at 37 °C to obtain CCA-M1EVs. Transmission electron microscopy (TEM) revealed that the M1EVs exhibited a classical cup-shaped morphology and had an average diameter of ~100 nm (Fig. 3a). Immunoblotting further verified that M1EVs expressed the small EV markers CD9, CD81, ALIX, TSG101, the macrophage marker F4/80, and the M1-macrophage marker iNOS (Fig. 3b). Successful modification with AQ4N or Ce6 was verified by confocal laser scanning microscopy (CLSM), as the signal of AQ4N (or Ce6) was highly co-localized with that of M1EVs (Fig. 3c). Flow cytometry analyses showed that 84% of the CCA-M1EVs were loaded with both AQ4N and TAMRA (used in place of Ce6 due to an overlapping spectrum with AQ4N) (Fig. 3d). The above data, combined with the absorbance spectra of AQ4N and Ce6 (Supplementary Fig. S6), confirmed the successful encapsulation of AQ4N and Ce6 into CCA-M1EVs. Subsequently, the mosaic of CPPO with CCA-M1EVs was verified by high-performance liquid chromatography (HPLC). The loading capacities of CPPO, Ce6, and AQ4N were calculated to be 7.3, 7.5, and 18.5 µg per 100 µg M1EVs. Nanoparticle tracking analysis (NTA) showed only negligible changes in the size distribution of EVs before and after drug loading (Supplementary Fig. S7a). No significant size changes in PBS were observed after 7 days (Supplementary Fig. S7b); thus, the EVs fulfilled the specific requirements for intravenous (*i.v*.) injection. In short, all of these data clearly indicated that loading M1EVs with cargoes had negligible influences on their original properties.

Next, we conducted multiple experiments to verify the function of the loaded components. 9,10-Anthracenediyl-bis(methylene)dimalonic acid (ABDA, an ROS indicator) was used to study ^1^O_2_ generation ability, and the Ce6/CPPO corresponding absorption intensities of ABDA at 399 nm decreased immediately after adding H_2_O_2_, supporting the efficient production of ^1^O_2_. CC-M1EVs and CCA-M1EVs exhibited similar ^1^O_2_ generation efficiencies as CPPO/Ce6, and the addition of AQ4N and M1EVs did not influence the ^1^O_2_ generation ability of the photosensitizer (Fig. 3e). Furthermore, to assess the ROS-triggered AQ4N release of CCA-M1EVs, we excited CCA-M1EVs upon the addition of H_2_O_2_ to trigger Ce6 to transform molecular oxygen into ^1^O_2_. As shown in Fig. 3f, the AQ4N concentration rapidly increased after the addition of H_2_O_2_, suggesting that ^1^O_2_ generated by CPPO/Ce6 could trigger the release of AQ4N. In addition, we confirmed that the reaction consumes oxygen, resulting in a decrease in the oxygen concentration, which indicated that CDT aggravates tumor hypoxia (Fig. 3g). As the concentration of O_2_ decreased, the ratio of AQ4/AQ4N increased, suggesting that hypoxia-triggered AQ4 generation for cell killing (Fig. 3h). Although the above evaluations were conducted separately in vitro, it should be noted that each reaction coupled in vivo and did not occur in a sequential manner.

Having successfully constructed the desired M1EVs delivery system, we next assessed BBB permeation ability of M1EVs loaded with different drugs using an in vitro model: a Transwell^TM^ co-culture system, in which bEnd.3 cells were cultured in the upper chamber and primary macrophages (MΦ) and GBM tumor cells (U87MG cells) were cultured in the lower chamber. M1EVs, A-M1EVs, CC-M1EVs, and CCA-M1EVs were added to the upper chamber (Fig. 3i). As shown in Fig. 3j, on the one hand, the integrity of the formed cell layer was confirmed by visualizing a well-known tight junction marker protein (ZO-1) by CLSM; on the other hand, EVs labeled with DiD could penetrate the BBB. Moreover, we calculated the fluorescence intensity of different EVs according to the fluorescence intensity of DiD in the lower chamber. The penetration efficiency of M1EVs increased with incubation time and eventually reached approximately 30% after 8 h, which again confirmed that M1EVs could cross the BBB model in vitro and that cargo loading had no effects on their penetration ability (Fig. 3k).

Having demonstrated that M1EVs could penetrate the BBB, we next evaluated the synergy among several components. We evaluated the modulatory effect of different formulations on the immune microenvironment by monitoring the M2/M1 ratio in the lower chamber, specifically by conducting flow cytometry analysis for M1 and M2 macrophages. The M2/M1 ratio was decreased in the M1EVs group compared with the PBS group, and this effect was maintained after loading of different drugs (Fig. 3l and Supplementary Fig. S8). Moreover, we noted that this M2-to-M1 polarization was accompanied by substantial increases in H_2_O_2_ levels: the H_2_O_2_ concentration was higher in all groups in which the microenvironment was modulated than in the PBS group (Fig. 3m). This is likely attributed to the increase in the proportion of M1 macrophages, which can activate the nicotinamide adenine phosphate dinucleotide oxidase system to induce ∙O_2_^-^ generation and further utilize the catalysis of superoxide dismutase for H_2_O_2_ production.^39,40^ Furthermore, we investigated CDT in the U87MG cellular environment using a DCFH-DA probe. Due to the presence of H_2_O_2_, CPPO/Ce6-containing groups (CC-M1EVs and CCA-M1EVs) generated a large amount of ROS (Fig.3n). In addition, we further investigated the apoptosis and cytotoxicity on U87MG cells in vitro using Annexin V/PI staining and the CCK8 analysis. As the above reaction consumed oxygen, aggravation of tumor hypoxia led to the conversion of AQ4N into AQ4; therefore, CCA-M1EVs treated cells exhibited significantly higher cell killing efficiency (Fig. 3o and Supplementary Fig. S9). Similarly, in this Transwell^TM^ system, CCA-M1EVs (mouse source) were also observed to have a potent killing effect on G422 and GL261 cells (Supplementary Fig. S10). Taken together, these results indicate that CCA-M1EVs have effective M2-to-M1 polarization, CDT, and hypoxia-activated chemotherapy, making it promising for synergistic cancer treatment.

### In vitro evaluation of the antitumor effects of CCA-M1EVs on multicellular tumor spheroids (MCTSs)

**Fig. 4:**
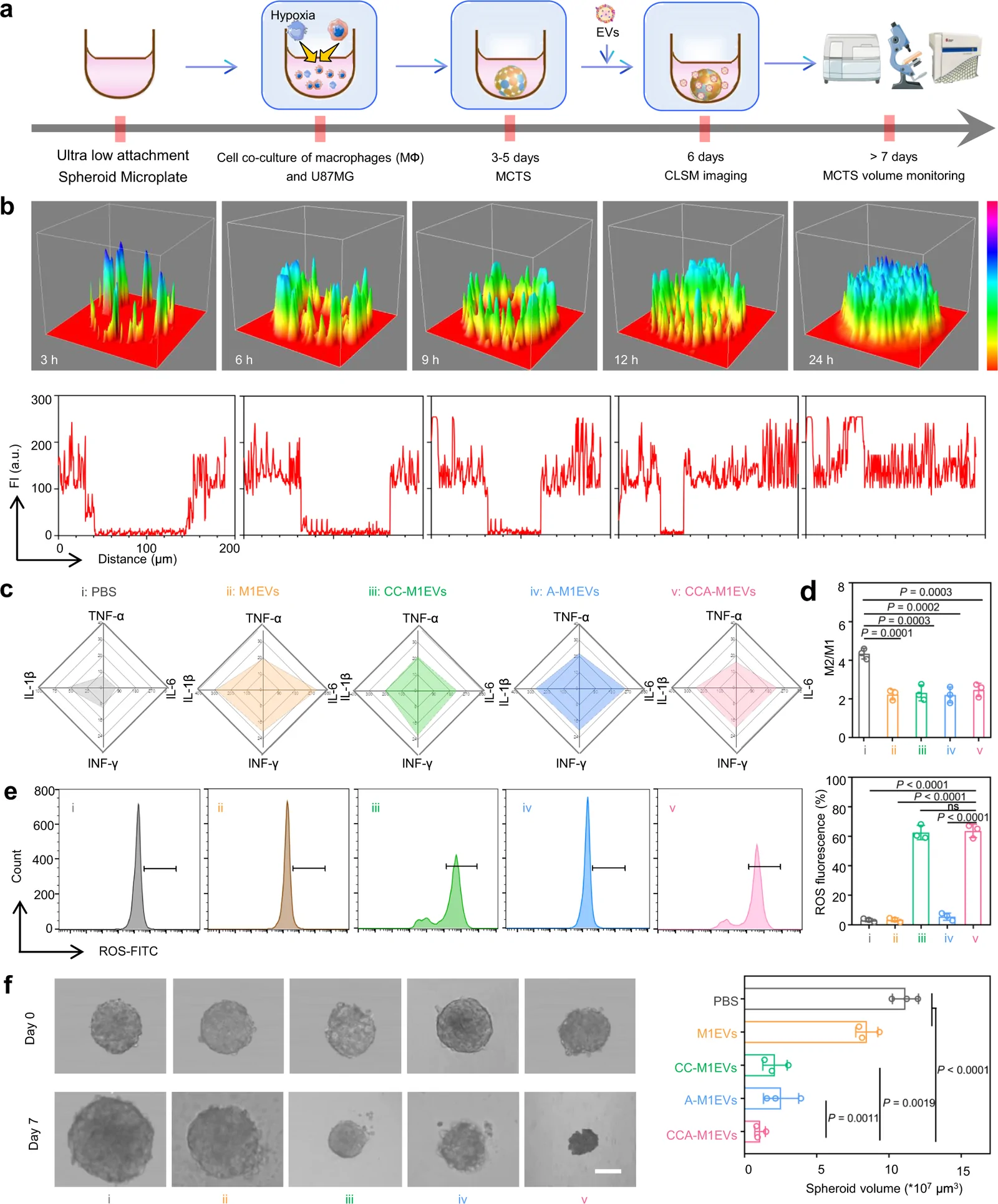
In vitro evaluation of the antitumor effects of CCA-M1EVs on multicellular tumor spheroids (MCTSs). **a**. Illustration of in vitro three-dimensional tumor model. **b**. CLSM images of surface plots of DiD-labeled M1EVs penetration in MCTS (top); the corresponding fluorescence signal intensity across the spheroids (bottom). **c**. The concentrations of IL-1β, IL-6, TNF-α, and IFN-γ in supernatants prepared from the MCTS culture medium after treatments with PBS, M1EVs, CC-M1EVs, A-M1EVs, and CCA-M1EVs, respectively. **d**. Quantitative analysis of M2/M1 ratio of the MCTS based on flow cytometry analysis (*n* = 3). **e**. Representative flow cytometry analysis images and corresponding quantitative analysis of the intracellular levels of ROS in U87MG cells treated with different EVs designs (*n* = 3). **f**. Photographs of MCTSs at a certain time (day 0 and day 7) (left). The volume of MCTSs treated with different formulations at day 7 (right) (*n* = 3). All images have the same scale of 100 μm. For **d**, **e**, and **f**, data are presented as the mean ± S.D. Statistical significance between multiple groups was calculated using one-way ANOVA with a Kruskal-Wallis test (**d**, **e**, and **f**). ns, not significant

Considering that two-dimensional (2D) cell culture models disregard the complexity of interactions seen in tumors, we conducted further experiments on MCTSs.^41,42^ Briefly, tumor spheroids were formed from U87MG cells and macrophages using the liquid overlay method (Fig. 4a).^43^ CLSM and corresponding line fluorescence intensity analysis of M1EVs into MCTS over a 24 h incubation showed time-dependent increases in the extent of M1EVs penetration into spheroids (Fig. 4b and Supplementary Fig. S11). Then, we further investigated their synergistic effects. The production of cytokines in the culture supernatant was measured with the Luminex multiplex cytokine analysis platform. Treatment containing M1EVs (M1EVs, CC-M1EVs, A-M1EVs, and CCA-M1EVs) efficiently increased the production of immune-active cytokines (TNF-α, IL-6, IFN-γ, and IL-1β) (Fig. 4c). All groups with elevated immune-active cytokine levels exhibited lower M2/M1 ratios (1.78–2.16) than PBS (~4.28), which demonstrated that M1EVs could modulate M2-to-M1 polarization (Fig. 4d). Moreover, we used the ROS probe DCFH-DA to monitor CPPO/Ce6-triggered singlet oxygen production in U87MG cells by flow cytometry analysis. Together with CPPO and Ce6 from CC-M1EVs and CCA-M1EVs, an abundance of ROS could be generated for CDT (Fig. 4e). Additionally, to assess the synergistic effect of chemotherapy and CDT after the addition of AQ4N, apoptosis of U87MG cells was analyzed by flow cytometry using Annexin V/PI staining. CCA-M1EVs exhibited the most potent cytotoxicity, which demonstrated that significantly enhanced anti-tumor efficiency could be achieved by CDT aggravated hypoxia to convert AQ4N into AQ4. The different formulations mentioned above, which had varying synergistic strategies, led to distinct degrees of inhibition. The final volume of spheroids decreased in the order of the PBS, M1EVs, A-M1EVs, CC-M1EVs, and CCA-M1EVs groups. Quantitatively, the spheroid treated with the CCA-M1EVs group with three synergistic effects shrank almost 60 times compared with the PBS group (Fig. 4f). Such potent MCTSs inhibition again reflected the importance of rational synergistic therapy for killing GBM.

### Therapeutic effects in a cell-derived xenograft model

**Fig. 5:**
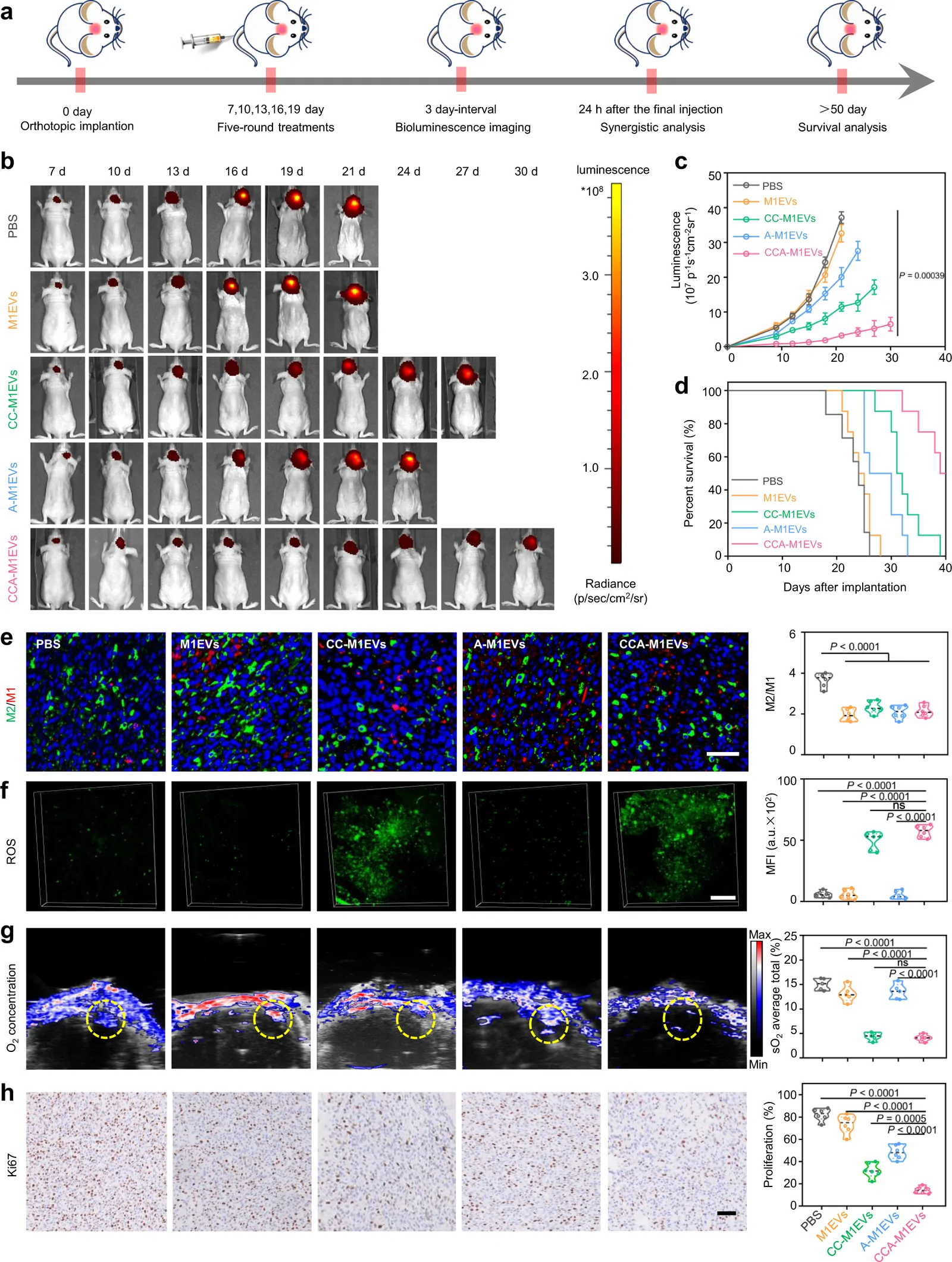
In vivo evaluation of the antitumor effects of CCA-M1EVs in U87MG-luc tumor-bearing mice. **a**. Experimental design for evaluating the efficiency of tumor inhibition upon treatments with PBS, M1EVs, A-M1EVs, CC-M1EVs, and CCA-M1EVs in U87MG-luc tumor-bearing model mice. The mice were given the indicated formulations at day 7, 10, 13, 16, and 19. Bioluminescence intensity in the brain was determined every three days using an IVIS III instrument. 24 h after the final injection, ROS production was detected by DCFH-DA using two-photon fluorescence images and O_2_ concentration was measured by photoacoustic (PA). Meanwhile, some of the mice in each group were sacrificed, and brains were harvested for TME and proliferation analyses. The remaining mice were used to monitor tumor growth and survival time. **b**. Representative bioluminescence images of U87MG-luc tumor-bearing mice after *i.v*. injection with different groups at the indicated time points. The blank area means mice were dead. **c**. Corresponding quantification of the total flux in luciferase signals from panel (**b**) (*n* = 8). **d**. Survival rate of the tumor-bearing mice upon treated with different groups (*n* = 8). **e**. Immunofluorescence imaging of brain histological sections of M2 (CD163, green) and M1 (iNOS, red), and corresponding quantification of M2/M1 ratio. All images have the same scale of 50 μm (*n* = 6). **f**. Two-photon fluorescence images of U87MG-bearing mice and quantitative analysis of ROS signals in tumor tissue after different treatments. All images have the same scale of 100 μm (*n* = 6). **g**. PA images of U87MG-bearing mice and quantitative analysis of the oxyhemoglobin saturation levels in tumors (*n* = 6). **h**. Ki67 staining of tumor sections for each group, with corresponding quantification on the right. All images have the same scale of 50 μm (*n* = 6). For **c**, **e**, **f**, **g**, and **h** are presented as the mean ± S.D. Statistical significance between multiple groups was calculated using one-way ANOVA with a Kruskal-Wallis test (**c**, **e**, **f**, **g**, **h**). Survival analysis was calculated with two-sided Log-rank Mantel-Cox tests (**d**). ns, not significant

The above results encouraged us to evaluate the therapeutic effects on U87MG-luc xenograft mice (Fig. 5a). At 7 days post-implantation, glioma-bearing nude mice were treated once every three days with different EV-based formulations. Compared with PBS, M1EVs exhibited a very slight antitumor effect through immunomodulation alone (Fig. 5b, c). In the A-M1EVs and CC-M1EVs groups, tumor growth was suppressed to various degrees. The greatest tumor inhibition and the smallest tumor volume were achieved in the CCA-M1EVs group due to synergistic of immunomodulation, CDT, and hypoxia-activated chemotherapy. Consistently, survival curves showed that the mice treated with CCA-M1EVs had markedly prolonged survival times (Fig. 5d), which were significantly longer than mice treated with A-M1EVs and CC-M1EVs. To evaluate safety in vivo, we performed hematoxylin and eosin (H&E) staining, assessed blood physiological and biochemical indices and measure the bodyweight of all treated mice; we observed no obvious physiological abnormalities or systemic toxicity (Supplementary Fig. S12-14). All these results indicate the apparent biosafety of our M1EVs-based delivery system.

To gain deeper insight into the inhibitory mechanism in vivo, we measured the synergistic effect of immunomodulation, CDT, and hypoxia-activated chemotherapy. Initially, immunochemical staining of M1-like and M2-like macrophages in tumor tissues indicated in vivo immunomodulatory activity with the number of M1-like macrophages (red fluorescence) being higher in the M1EVs-derived (M1EVs, CC-M1EVs, A-M1EVs, and CCA-M1EVs) groups than in the PBS group (Fig. 5e). Moreover, compared with PBS, M1EVs, and A-M1EVs, treatments containing CPPO and Ce6 (CC-M1EVs and CCA-M1EVs) efficiently increased the level of ROS, as observed by two-photon fluorescence microscopy (Fig. 5f). Furthermore, oxygenation levels throughout the entire tumor area (sO_2_ AVT Total) were decreased at 24 h post-injection of CC-M1EVs and CCA-M1EVs, which was detected by photoacoustic (PA) imaging. No similar changes in oxygenation levels were detected for mice *i.v*. injected with PBS, M1EVs, or A-M1EVs (Fig. 5g). Tumor cellular proliferation was observed by staining for Ki67 (Fig. 5h), and the results further confirmed that the treatment exerted synergetic immunomodulation, CDT, and hypoxia-activated chemotherapy with an amplified antitumor efficacy.

### Construction of human M1-macrophage based formulation and verification of efficacy in a PDX model

**Fig. 6:**
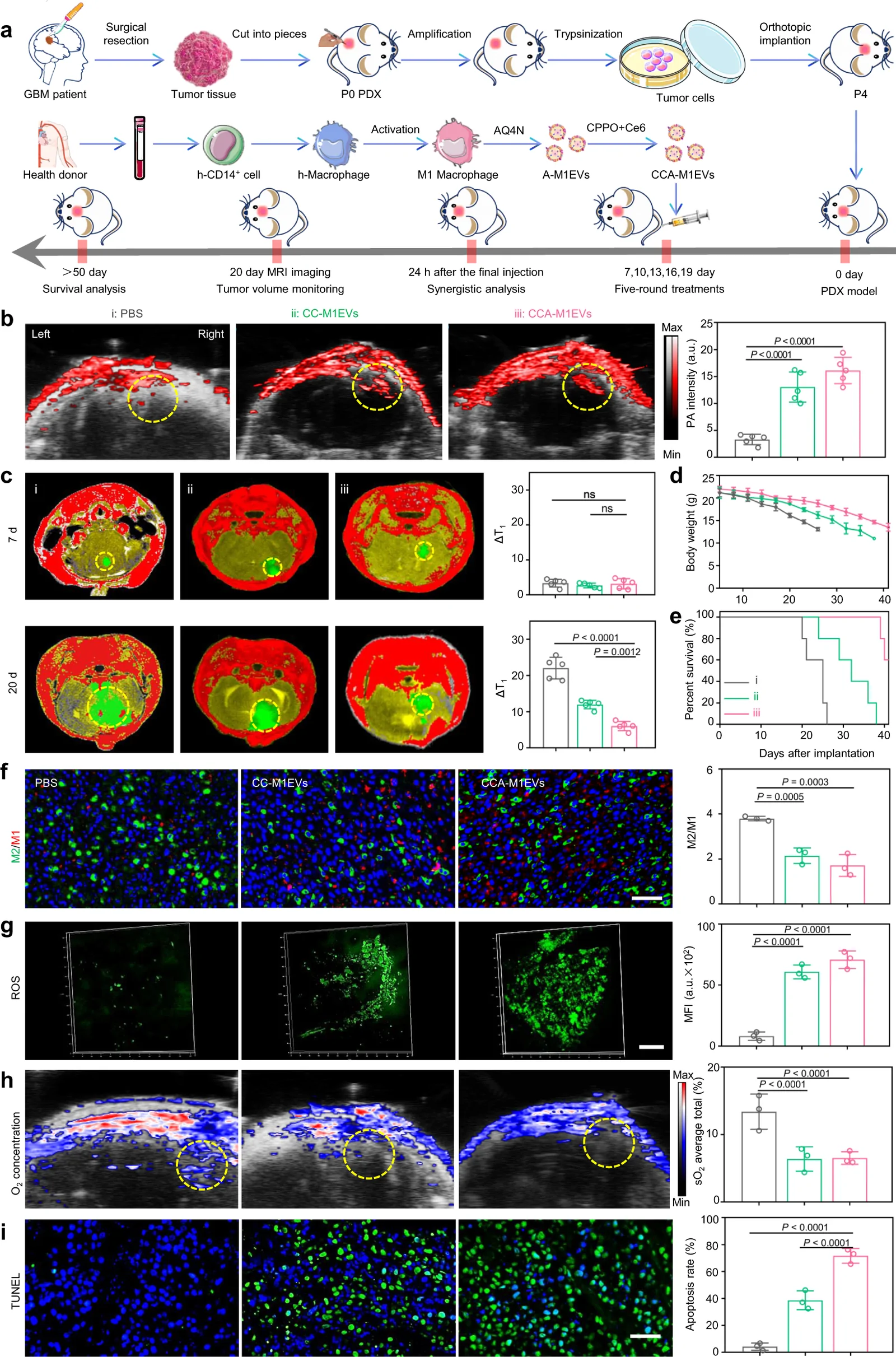
CCA-M1EVs exhibited potent anti-tumor effects against patient-derived xenograft (PDX) model in vivo. **a**. Schematic illustration of PDX model, humanized EVs construction, and experimental design for evaluating the efficiency of tumor inhibition upon treatment with PBS, CC-M1EVs, and CCA-M1EVs (human PBMC source) in PDX mice. The mice were given the indicated formulations at day 7, 10, 13, 16 and 19. T_1_-weighted MR signals in the brain was determined using magnetic resonance imaging (MRI) at day 7 and 20. 24 h after the final injection, ROS production was detected by DCFH-DA using two-photon fluorescence images and O_2_ concentration was measured by PA. Meanwhile, some of the mice in each group were sacrificed, and brains were harvested for TME and terminal deoxynucleotidyl transferase-mediated dUTP-biotin nick end labeling (TUNEL) analyses. The remaining mice were used to monitor survival time. **b**. In vivo PA images and relative PA signal intensity statistics of GBM PDX mice after *i.v*. injection of PBS, CC-M1EVs, or CCA-M1EVs, respectively. Here, Ce6 was served as a PA signals for the assessment of the distribution of CC-M1EVs and CCA-M1EVs (*n* = 5). **c**. T_1_-weighted MRI of GBM PDX tumor-bearing mice at 7 day and 20 day post *i.v*. injection with various groups, and corresponding quantification of T_1_-weighted MRI from the tumor site (*n* = 5). Images were analyzed with Analyze 11.0. **d**. The body weight of the mice with different treatments (*n* = 5). **e**. Varieties of survival rates of PDX tumor-bearing mice in different groups (*n* = 5). **f**. Immunofluorescence imaging of brain histological sections of M2 (CD163, green) and M1 (iNOS, red), and corresponding quantification of M2/M1 ratio. All images have the same scale of 50 μm (*n* = 3). **g**. Two-photon fluorescence images of GBM PDX tumor-bearing mice and quantitative analysis of ROS signals in tumor tissues after *i.v*. injection different treatments. All images have the same scale of 100 μm (*n* = 3). **h**. PA images of GBM PDX tumor-bearing mice and quantitative analysis of the oxyhemoglobin saturation levels in tumors with treatment of different extracellular vesicle designs (n = 3). **i**. TUNEL staining of tumor sections for each group, with corresponding quantification (*n* = 3). All images have the same scale of 50 μm. For **b**, **c**, **d**, **f**, **g**, **h**, and **i**, data are presented as the mean ± S.D. Statistical significance between multiple groups was calculated using one-way ANOVA with a Kruskal-Wallis test (**b**, **c**, **d**, **f**, **g**, **h**, and **i**). Survival analysis was calculated using two-sided Log-rank Mantel-Cox tests (**e**). ns, not significant

To further investigate the potential of our M1EVs platform, we evaluated its therapeutic effect in a PDX model (Fig. 6a). Briefly, a primary tumor sample was resected from a GBM patient. This tumor sample was subcutaneously (*s.c*.) transplanted into the axillae of the nude mice. After engraftment for three passages, the tumor tissue was dissociated into single cells by treatment with trypsin. Then, the tumor cells were transplanted into the brains of mice to establish PDX models,^44–46^ which had the characteristics of primary tumors, as observed by H&E staining (Supplementary Fig. S15). The successful preparation of M1EVs (from human PBMCs) and the establishment of an intracranial PDX model thus paved the way for subsequent investigation.

After intravenous injection, PA imaging was used to monitor the bio-distribution of different formulation (CC-M1EVs and CCA-M1EVs) in the brain due to the photoacoustic performance of Ce6. PA signals were identified at 12 h post-injection, and apparent signals were observed at the tumor site (Fig. 6b), indicating that CC-M1EVs and CCA-M1EVs could successfully accumulate in tumors. Subsequently, magnetic resonance imaging (MRI) was used to assess the anti-tumor effect. Compared with those in the PBS group, mice treated with CC-M1EVs showed moderated tumor growth inhibition, while the CCA-M1EVs group showed further enhancement of tumor inhibition (Fig. 6c and Supplementary Fig. S16). As a result, excellent tumor growth inhibition was achieved with delayed body-weight loss (Fig. 6d) and a prolonged survival time (Fig. 6e). To explore the mechanism underlying the observed tumor growth inhibition, we then conducted a series of experiments in the PDX tumor model. Compared with PBS, CC-M1EVs and CCA-M1EVs efficiently modulated M2-to-M1 polarization (Fig. 6f), increased the level of ROS (Fig. 6g), and obviously decreased the concentration of O_2_ (Fig. 6h). Similar findings regarding the greatest effects on the enhancement of immunomodulation, CDT, and hypoxia-activated chemotherapy in the CCA-M1EVs group were observed by terminal deoxynucleotidyl transferase-mediated dUTP-biotin nick end labeling (TUNEL) staining of tumor slices from CDX model mice (Fig. 6i). These important physiologies revealed few abnormalities (Supplementary Fig. S17), which further confirmed the safety of our nanoplatform. These data suggest that our CCA-M1EVs platform provides safe, specific, and highly effective antitumor efficacy in a PDX model.

## Discussion

There are multiple challenges that make GBM a particularly deadly and difficult-to-treat cancer, mainly including the problems from the contact BBB and complex physiological conditions.^28^ Therefore, a chasm exists in the field of oncology between research on GBM and that on other solid tumors. Although many new modalities have been developed for the treatment of other solid tumors, they may not be applicable for the treatment of GBM. The development of efficient targeted delivery vectors and potent therapeutic modalities is still a primary concern in GBM research. By developing a flexible system based on M1-macrophage-derived extracellular vesicles, we showed how simultaneously exploiting multiple vulnerabilities and cellular processes could result in powerful effects against GBM cells and CDX and PDX tumors. Our examinations of clinical samples initially revealed that the human GBM TME was rich in M2 macrophages and featured extensive infiltration of macrophages derived from peripheral blood. These two new findings inspired us to develop the M1EVs platform and the eventual development of CCA-M1EVs. Future efforts can be devoted to investigating the infiltration of M2 macrophages and the source of these cells based on gliomas of other molecular classifications (such as IDH mutation, promoter methylation of MGMT, chromosomal deletion of 1p/19q, B-RAF fusion, and point mutation), which can provide guidelines for more rational and precise utilization of our CCA-M1EVs in the treatment of GBM.

Among the three synergistic modalities, CDT was based on the utilization of CPPO and Ce6 as a testbed, which could overcome the PDT-associated restriction of the depth of light penetration. Although the efficacy of this modality for the treatment of other solid tumors has been explored, it has not been found to be a silver bullet for the treatment of malignant GBM. In addition to aforementioned contact BBB, current dilemmas of using CDT for GBM therapy also include the inefficient intertumoral H_2_O_2_, and rare synergism with other therapeutic modalities. Herein, we provide the first example a rationally designed system using CPPO and Ce6 for the efficient treatment of GBM. Upon application of macrophage-derived EVs as targeted carriers for efficient delivery to GBM, the increase in intratumoral H_2_O_2_ resulting from M1EVs-induced immunomodulation and AQ4 activation resulting from oxygen consumption during ROS production achieved synergism with CDT, resulting in satisfactory therapeutic outcomes.

These findings clearly highlight the strong potential of our M1EVs system to be developed into new therapies for GBM. As many tumor types are known to feature M2-TAM infiltration, it should also be emphasized that our CCA-M1EVs can be seen as potential agents for treating other tumors, such as lung cancer, gastric cancer, and pancreatic cancer. In addition to AQ4N and CPPO/Ce6, it should be possible to flexibly load highly diverse cargo combinations into the inner cavity and the outer shell of our CCA-M1EVs to meet diverse requirements and achieve successful synergistic efficacy against many tumor types. Besides cancers, there have been reports on large-scale macrophage infiltration in other brain diseases (e.g., ischemic stroke); thus, macrophage-derived EVs may also represent promising agents for the treatment of other brain diseases. To facilitate the translation of these EV-based therapies into the clinic, more efforts should be made in their mass production. One possible method is the genetic engineering of cells to induce the overexpression of activator genes (i.e., HSP20, TSPAN6, CD9) of EV biogenesis.^47^ Alternatively, the cell culture medium can also be altered (i.e., via glucose starvation, an acidic pH, or shear stress) to force cells to produce more EVs.^48^ Given that the above manipulations may also alter EVs composition, the safety and therapeutic effects of these EVs should be evaluated and confirmed.

## Materials and Methods

### Chemicals and materials

Bis(2,4,5-trichloro-6-carbopentoxyphenyl) oxalate (CPPO), Ficoll, DCFH-DAm and lipopolysaccharides (LPS) were obtained from Sigma-Aldrich (St. Louis, MO, USA). Alexa Flour^TM^-488-phalloidin and TAMRA were purchased from Thermo Fisher Scientific (Massachusetts, USA). DiR, DiO, DiD, propidium iodide, fluorescein isothiocyanate (FITC), 6-diamidino-2-phinylindolo dihydrochloride (DAPI)m and Cyanine 7 (Cy7) were obtained from Fanbo Biochemical (Beijing, China). AQ4N was purchased from MedChemExpress. Anti-CD9, anti-CD81, anti-ALIX, anti-TSG101, anti-Ki67, anti-iNOS, anti-CD163, anti-TMEM119, anti-GADPH, and anti-ZO-1 were purchased from Abcam (Cambridge, England). Anti-F4/80 was purchased from CST. GM-CSF was purchased from eBiosciences. Small EVs Spin Columns were obtained from Invitrogen Co. (California, USA). PEG-PLGA NPs was synthesized in our laboratory as previously described. Other chemicals were purchased from J&K (Beijing, China).

### Cell culture

The mouse brain endothelial cells (bEnd.3) were supplied by the American Type Culture Collection (ATCC). The luciferase-transfected glioblastoma cell lines (U87MG-luc, GL261-luc, and G422-luc) were maintained in our laboratory (Shenzhen Second People’s Hospital, Shenzhen, China). Both types of cells were cultured in DMEM medium (Gibco BRL) containing 10% fetal calf serum (Gibco BRL), streptomycin (100 μg/mL, Sigma-Aldrich) and penicillin (100 U/mL, Sigma-Aldrich) at 37°C with 5% CO_2_, and all the cells were passaged at approximately 80% confluency.

### Animals

Female BALB/c nude mice (6–8 weeks, 18–22 g), female Kunming mice (4–6 weeks), and male C57BL/6 mice (6–8 weeks, 18–22 g) were purchased from Beijing Vital River Laboratory Animal Technology Co., Ltd. (Beijing, China), and all animals were kept in IVC mouse cages with standard conditions and free access to food and water. The animal protocol was approved by the Institutional Animal Care and Use Committees at the Institute of Process Engineering, Chinese Academy of Sciences (approval ID: IPEAECA2021103). All animal experiments was performed in accordance with the Guide for the Care and Use of Laboratory Animals (China, GB/T 35892-2018).

### Patient samples, isolation of peripheral blood mononuclear cells (PBMCs), and construction of PDX model

Blood and brain tumor specimens were obtained from patients with informed consent and were reviewed by the pathologist and surgeon. Pathologist classified the type and grade of the tumors in accordance with the WHO histological grading of central nervous system tumors. Sixty-four cases of gliomas were selected from Shenzhen Second People’s Hospital. For IHC, tumor-adjacent tissue was taken as a control. We confirmed tumor-adjacent tissue through histopathology. Characteristics of glioma patients were shown in Supplementary Table S1. Healthy volunteers were recruited through the protocol at the Shenzhen Second People’s Hospital. Peripheral blood samples were acquired from healthy volunteers (age 25–30, male).

Isolation of PBMC: human whole blood was collected in heparin tubes and PBMCs were separated by Ficoll-hypaque density gradient centrifugation (BD, SanDiego, CA). Then PBMC were separated to CD14^+^ monocytes with corresponding magnetic beads. The CD14^+^ monocytes differentiated to macrophage under 50 ng/mL GM-CSF treatment and further polarized to M1-like macrophage under LPS (1 µg/mL) treatment.

Construction of PDX model:^49^ transport tumor sample from pathology to laboratory in HBSS at room temperature and record relevant patient information (e.g., age, sex, etc) (Supplementary Table S2). Then the tumor sample was subcutaneous transplanted into the axilla of the nude mice (female, 6–8 weeks old). After engraftment for three passages, tumor tissue was dissociated into single cells by treatment with trypsin-EDTA digestion. The cells (10^5^ cells) were stereotactically injected into the brain parenchyma at a depth of 3 mm.^50^ At 2 weeks after the injection of the tumor cells, thin sections of the mouse brain (4 μm) were processed for H&E staining. All procedures performed in studies involving human participants were in accordance with the ethical standards of the institutional committee. The study was approved by Ethics Committee of Shenzhen Second People’s Hospital Clinical trials (20200727004-FS01).

### Orthotopic transplantation of glioma cells

After deep anesthesia, C57BL/6 mice (or Kunming) were positioned in a stereotactic frame (RWD, Shenzhen, China). Punch a small hole with a 25-gauge needle behind right bregma and 2.5–3 mm away from the midline. Then, the GL261-luc cells (or G422-luc) (10^5^ cells) were stereotactically injected into the brain parenchyma at a depth of 3 mm.

### Immunofluorescence assay and Immunohistochemistry

Four-μm-thick tissue sections (human and mice) were de-waxed in rehydrated through graded alcohols. Antigen retrieval was carried out using Dako PT link (Dako/Agilent Technologies, Santa Clara, CA). IHC staining of individual markers iNOS, CD163 or Ki67 was performed using EnVision™ G | 2 Doublestain System, rabbit/mouse (DAB/Permanent Red) kit (Dako/Agilent Technologies, Santa Clara, CA), according to the manufacturer’s instructions. IF staining of iNOS (or CD163) and TMEM119 was performed according to the manufacturer’s instructions. Slices were imaged using Vectra II Polaris Automated Quantitative Pathology Imaging System, and images were analyzed with inForm 2.4.

### Database

cBioportal (URL: http://www.cbioportal.org/) was used to assess the dataset of LGG and HGG from TCGA.^51^ Freely accessible server, oncolnc tool (URL: www.oncol.nc.org) was used for analyzing the survival correlation of selected M1 and M2 macrophage marker.^52^ OncoLnc tool generated the Kaplan-Meier plots for the studied genes using the low and high-expressing M2/M1 ratio that are publically available in TCGA database.

### Isolation and extraction of M0EVs, M1EVs, M2EVs, and EMVs

C57/BL6 mice were intraperitoneal injected with 6% starch broth to induce inflammatory responses and elicit large numbers of macrophages. We harvested starch broth-elicited peritoneal cells and cultured them in dishes (10^6^ cells per pore of 12 microwell plate). Pure adherent macrophages would be separated from other types of cells in the peritoneal cavity. Macrophages were treated with or without 1 μg/mL LPS. Cell cultures were EV-depleted media prepared by ultracentrifugation of FBS for 3 h at 200,000 g. After 48 h treatments, cell culture supernatant was collected. EVs were prepared according to a typical protocol.^53,54^ Briefly, culture supernatant was centrifuged at 300 × *g* or 2000 × *g* for 10 min to remove cells and cell fragments, respectively. Then the obtained supernatant was centrifuged at 10,000 g for 30 min at 4 °C to remove debris. The final supernatant was then ultracentrifuged at 100,000 × *g* for 70 min twice to obtain a pellet containing M1EVs (mouse). The same method was used to obtain human PBMC M1EVs.

The whole blood of C57 mice was harvested by retro-orbital puncture and collected in heparinized mouse blood collection tubes. The blood was centrifuged at 3000 rpm, and the red blood cells were placed in a 3-fold amount of precooled isotonic phosphate buffer (pH 7.4). Then centrifugation at 5000 rpm×15 min, added 10 mmol/L low permeability Tris-HCl buffer solution, and placed in 4°C refrigerator for 1–2 h, 4°C 15 min at 9000 rpm. Red corpuscles (100 nm) were prepared by mini extruder (EMVs).^55^

### In vivo and ex vivo imaging

M1EVs and EMVs were fluorescently labelled by incubation with 1 µM DiR solution, and excess dye was removed by either centrifugation or ultrafiltration for three times. The orthotopic glioma model using luc-U87MG cells were established in the study. The mice were anesthetized by intraperitoneal injection of pentobarbital sodium (1%). Then, cells (10^5^ cells) were inoculated into the right striatum (2.5 mm from the midline, 3.0 mm anterior to the bregma, and 3.0 mm deep) of nude mice using a stereotactic fixation device with mouse adaptor (RWD Life Science, Shenzhen, China). Then the scalp was closed with a clip. In vivo fluorescence imaging was initially used to evaluate the biodistribution and targeting efficacy of nanoplatform on orthotopic GBM models. After seven days of feeding, the orthotopic GBM-bearing mice were i.v. injected with M1EVs, EMVs, and PEG NPs (labelled with DiR). The mice were observed at indicated time points (1, 3, 6, 9, 12, and 48 h) after injection using an IVIS imaging system (PerkinElmer, USA). After 48 h, the mice in the three groups were sacrificed, and the brain, heart, liver, spleen, lung, kidney, and intestine were taken for observation and analysis. Ex vivo images as described above were also recorded. Frozen sections of tumor at 48 h were prepared, and detected by automatic multispectral imaging system (PerkinElmer Vectra II) after DAPI staining.

The aggregation behavior of particles in vivo was also tested by a two-photon laser confocal scanning fluorescence microscope (labelled with DiO or FITC, tested by Leica TCS SP8, Germany). To fashion a cranial window, the skull was thinned away using a sterile stainless steel 2 mm diameter cylindrical drill bit attached to a high-speed hand drill until the underlying dura mater was exposed.

### Synthesis of CCA-M1EVs

M1 macrophages were treated with LPS (1 μg/mL) and AQ4N (100 μM), after 48 h treatment, macrophages and cell culture supernatant were collected. At the same time, A-M1EVs were isolated from macrophages supernatant by ultracentrifugation. Then, we added 100 µg CPPO (dissolved in 5 µl THF) and 120 µg Ce6 (dissolved in 5 µl DMSO) in 200 µg A-M1EVs (dispersed in 1 ml PBS). After 1 h incubation, the resulting CCA-M1EVs were washed with PBS for three times to remove the free CPPO and Ce6. The unloaded drugs were removed by elution with a 100 kDa ultrafiltration tube (Merck Millipore Co., Darmstadt, Germany).

### Characterization of CCA-M1EVs

Transmission electron microscope (TEM) samples of EVs were prepared according to a typical protocol,^56^ and imaged by the HITACHI HT7700 TEM. The expression of CD9/CD81/ALIX/TSG101 (EV marker), iNOS (M1 marker) and F4/80 (macrophages marker) in M1 macrophage and M1EVs were analyzed by ProteinSimple® Wes^TM^ capillary western blot analyzer (PS-MK15; ProteinSimple, USA). Briefly, total protein of EVs was quantified using the BCA assay kit. EVs extracted from same amounts of cells were diluted (1:2) with sample buffer (ProteinSimple) and the quantification was performed using a 12–230 kDa 25-lane plate (PS-MK15, ProteinSimple) in WES according to the manufacturer’s instructions. CLSM and flow cytometry were used to investigate the colocalization of AQ4N, Ce6, and DiO- labeled M1EVs. Owing to AQ4N and Ce6 share the same spectrum, Ce6 was replaced with TAMRA in the flow cytometry analysis. Drug loading efficiency of AQ4N/Ce6 on CCA-M1EVs was detected by the microplate reader (AQ4N A_610 nm_, ε=22.5 mL/mg/cm; Ce6 A_404 nm_, ε=161 mL/mg/cm) (Tecan Infinite M200). Drug loading efficiency of CPPO on CCA-M1EVs was detected by HPLC (Agilent, USA). An Agilent-C18 column (5 µm particles, 4.6×250 mm) was used, and acetonitrile was used as the mobile phase at a flow rate of 1 mL/min. The UV absorbance was determined at 220 nm, and the column temperature was 25 °C.

The particle size and zeta potential of M1EVs before and after drug loading was measured by nanoparticle tracking analysis (NTA; Zetaview, Particle Metrix) at 25 °C. The CCA-M1EVs were dispersed in water or PBS, and then, the zeta potential and diameter were measured every day over the following one week by NTA. The ability of Ce6, CPPO/Ce6, CC-M1EVs, and CCA-M1EVs to generate chemiexcited ROS was evaluated using ABDA as an indicator. The measurement of ROS production of different formulations by microplate assay, followed by addition of different concentrations of H_2_O_2._ In the presence of H_2_O_2_, drug release behavior of AQ4N from CCA-M1EVs was detected by the microplate reader. The measurement of O_2_ consumation of different formulations by multiparameter analyzer (Mettler Toledo, Shanghai, China). The measurement of AQ4/AQ4N ratio of different formulations under hypoxia conditions measured by HPLC. An Agilent-C18 column (5 µm particles, 4.6×250 mm) was used with mobile phase of acetonitrile-ammonium formate buffer (0.05 M) (22:78, *v/v*), with final pH adjusted to 3.6 with formic acid. The UV absorbance was determined at 242 nm, and the column temperature was at 25 °C.

### Chemotactic migration across the BBB

The in vitro BBB model was constructed with bEnd.3 cells using a Transwell^TM^ cell culture system. Briefly, bEnd.3 cells (1×10^4^ cells/well) were seeded onto the upper chamber of the Transwell^TM^ pre-coated with gelatine (2% w-v) in 24-well plates, and primary macrophages and GBM tumor cells (U87MG) (1×10^3^ cells/well) were cultured in the lower chamber. After incubation for several days, the integrity of the cell monolayer was examined by measuring the tight junctional protein (ZO-1) using CLSM. Then, DiD-labeled EVs (~50 μg) in fresh culture media was added to the upper chamber. The penetration efficiency was determined by collecting samples from the lower chamber at the time points of 1, 2, 3, 4, 5, 6, and 8 h. The concentration of different formulations in the lower chamber was analyzed based on DiD fluorescence determined in a spectrofluorometer using excitation at 644 nm and emission at 665 nm.

Neutralizing antibodies against CCR2 (CXCR3, or CX3CR1) (Abcam, Cambridge, England) were used in antibody-blocking experiments. Then, DiD-labeled M1EVs (M0EVs) were pre-incubated with 10 µg/ml anti-CCR2 (anti-CXCR3, or anti-CX3CR1) antibodies for 30 min before added to the upper chamber. The penetration efficiency was determined by collecting samples from the lower chamber at 8 h. Based on DiD fluorescence to analyze the concentration of different formulations in the lower chamber.

The lower Transwell^TM^ chamber of M2 (F4/80 + CD163 + )/M1 (F4/80+iNOS + ) ratio was analyzed by flow cytometry. Single-cell suspensisons were subsequently stained with fluorescent antibody. The cells were then washed and analyzed using CytoFLEX LX Flow Cytometry. The H_2_O_2_ concentrations in the Transwell^TM^ lower chamber were measured using Hydrogen Peroxide Assay Kit according to the manufacturer’s instructions. The U87MG/MΦ cells were incubated with anti-CD11b magnetic beads and U87MG cells were obtained using the MACS cell sorting system protocol (Miltenyi Biotec., Germany). The intracellular ROS level of U87MG cells was detected by flow cytometry using the fluorescent probe DCFH-DA. The ROS were reacted with DCFH-DA for 20 min at 37 °C. Non-fluorescent DCFH can be converted to fluorescent DCF by ROS oxidation. The cellular ROS oxidized DCF can be used as indicator for ROS production (ex 488, em 510–555 nm). Cell apoptosis of CCA-M1EVs to U87MG cells under hypoxia conditions measured by flow cytometry using the Annexin V FITC/PI kit, according to the manufacturer’s instructions. Cell cytotoxicity of CCA-M1EVs to U87MG cells was measured by CCK8 (Beyotime Co., Shanghai, China). After 24 h incubation, CCK-8 solution was added and incubated for another 4 h. Percent viability was normalized according to the untreated cells.

### Penetration and growth inhibiton of MCTSs in vitro

Tumor spheroids of U87MG and macrophage cells (3:1) were prepared using the liquid overlay methods. To evaluate drug penetration in MCTS, cell spheroids were incubated with M1EVs for 24 h, and then analyzed by CLSM. The MCTS were incubated with different treatments, including PBS, M1EVs, CC-M1EVs, A-M1EVs, and CCA-M1EVs (~50 μg). At the end of the culture, each supernatant was collected and cytokines content was measured by Luminex multiplex cytokine analysis platform. The M2/M1 ratio was analyzed by flow cytometry. The U87MG/MΦ cells were incubated with anti-CD11b magnetic beads and U87MG cells were obtained using the MACS cell sorting system protocol. The intracellular ROS level of U87MG cells was detected by flow cytometry using the DCFH-DA. Under hypoxic conditions, the apoptosis of U87MG cells was detected by Annexin V-FITC and PI.

To estimate the growth inhibition effect on multicellular tumor spheroids, growth inhibition of the tumor spheroids was monitored using an inverted phase microscope. The major (r_max_) and minor (r_min_) radii of each treated MCTS were determined, and the spheroid volume was calculated according to equation 1:

1 $${{{\mathrm{V}}}} = \left( {{{{\mathrm{3}}}}/{{{\mathrm{4}}}}} \right) \times \pi \times \left( {{{{\mathrm{r}}}}_{{{{\mathrm{max}}}}}/{{{\mathrm{2}}}} + {{{\mathrm{r}}}}_{{{{\mathrm{min}}}}}/{{{\mathrm{2}}}}} \right)^{{{\mathrm{3}}}}$$

### Therapeutic effect and toxicity in vivo

For investigating antitumor effect of different treatments, U87MG-derived tumor xenografts were generated as described above, and the growth of orthotopic GBM could be monitored by the assistance of bioluminescence imaging. The glioma-bearing mice were given *i.p*. injections of a D-luciferin potassium solution (3 mg/mouse). Photons emitted from the glioblastoma region were collected and quantified by using the living image software. All images were treated with the same conditions and color scale. U87MG glioma-bearing mice were randomly divided into five groups (for each group, *n* = 8): PBS, M1EVs, CC-M1EVs, A-M1EVs, and CCA-M1EVs (~200 μg). Then, 100 μL of different formulations were intravenously injected every three days for 5 times after 7 days. Mice were regularly measured for any signs of deterioration or weight loss, and the body-weight and survival time of each mouse were recorded daily during the whole period of treatment.

To estimate the ROS production in tumors in situ, DCFH-DA was intratumorally administered and further the tumor was imaged by two-photon confocal scanning microscopy. Hypoxia tendency in vivo in tumors was measured by PA imaging. Specifically, the tumor oxygenation status was detected by the ratios of oxygenated hemoglobin (λ = 850 nm) and deoxygenated hemoglobin (λ = 750 nm) after *i.v*. injection of different EV formulations. At predetermined time points, animals were sacrificed and tumors were harvested. Then, the tumors were embedded in OCT medium. Tumor slices were stained by M2 (green), M1 (red), and Ki67 markers, respectively slides were scanned with automatic multispectral imaging system (PE Vectra II) and images were analyzed with inForm 2.4. To further evaluate the safety of different formulations in vivo, body-weight changes were recorded.

### Photoacoustic imaging and magnetic resonance in the PDX model

The orthotopic PDX-bearing mice were established as described above, and the tumor was determined using an in vivo imaging system (BioSpec 70/20 USR, Bruker, Germany). Then, the PDX mice were individually *i.v*. injected with CC-M1EVs and CCA-M1EVs (n = 5 per group) on days 7, 10, 13, 16, and 19. At 24 h post-injection, PA imaging was used to monitor the biodistribution of different formulations in the brain by Vevo LAZER (VisualSonics FujiFilm, Canada) due to the photoacoustic performance of Ce6.

Tumor progression was assessed by MRI at 7 day and 20 day. MRI imaging T_1_-weighted Gd contrast-enhanced (T_1_Gd) image regions allowed approximate delineation of tumor. The body weight and survival were measured every 3 days.

### Statistics

All statistical calculations were conducted using GraphPad Prism 9.0.0. Data presentation, sample size, and probability values were indicated in figure legends. For comparison between groups, statistical significance was done using unpaired two-tailed Student’s *t-*test. One-way ANOVA with a Tukey post hoc test (or Kruskal-Wallis test) was used for multiple-group comparisons. Survival analysis was calculated by two-sided Log-rank Mantel-Cox tests. *P* values < 0.05 were considered as significant, and *ns* indicated no significant difference.

## Supplementary information

Supplemental material

## Data Availability

All data or resources used in the paper are available by reasonable requirements to the leading correspondence, Prof. Wei Wei (weiwei@ipe.ac.cn).
