# Correction: Exploration and functionalization of M1-macrophage extracellular vesicles for effective accumulation in glioblastoma and strong synergistic therapeutic effects

**DOI:** 10.1038/s41392-026-02976-y

**Published:** 2026-09-05

**Authors:** Xiaojun Wang, Hui Ding, Zongyang Li, Yaonan Peng, Hui Tan, Changlong Wang, Guodong Huang, Weiping Li, Guanghui Ma, Wei Wei

**Affiliations:** 1 Shenzhen Key Laboratory of Neurosurgery, Department of Neurosurgery, Shenzhen Second People’s Hospital, Shenzhen University 1st Affiliated Hospital, Guangdong, China; 2 State Key Laboratory of Biochemical Engineering, Institute of Process Engineering, Chinese Academy of Sciences, Beijing, China; 3 Department of Ophthalmology, Beijing Chaoyang Hospital, Capital Medical University, Beijing, China; 4 Department of Otolaryngology and Institute of Translational Medicine, Shenzhen Second People’s Hospital, the First Affiliated Hospital of Shenzhen University Health Science Center, Shenzhen, Guangdong, China; 5 Department of Neurosurgery, Jinling Hospital, Jiangsu, Nanjing, China; 6 School of Chemical Engineering, University of Chinese Academy of Sciences, Beijing, China

Correction to: *Signal Transduction and Targeted Therapy* https://doi.org/10.1038/s41392-022-00894-3, published online 16 March 2022

Following online publication of article,^1^ unintentional error was identified in Fig. 1b and Supplementary Fig. S13. These errors have now been corrected, and the updated figures are provided below. Importantly, this correction does not affect the statistical analyses, conclusions, or overall integrity of the study.

In Fig. 1b, an incorrect CD163 immunohistochemistry (IHC) image was used for the anaplastic astrocytoma group, and an incorrect Ki67 IHC image was adopted for the diffuse astrocytoma group. This oversight occurred during data sorting and figure assembly, leading to the inadvertent incorporation of IHC images. The corrected figures are shown below.

Incorrect Fig. 1b:

**Figure:**
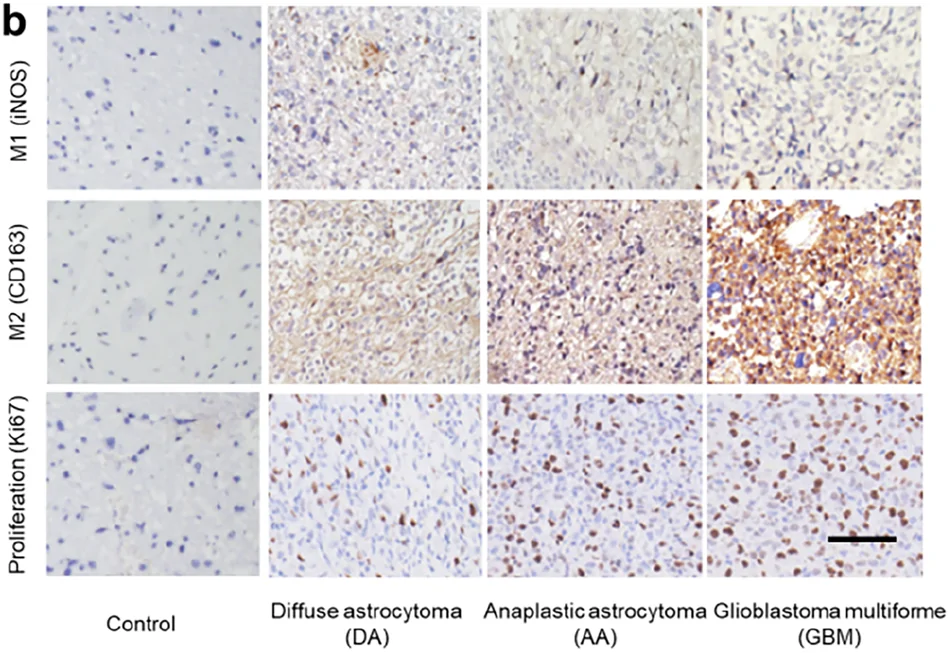

Correct Fig. 1b:

**Figure:**
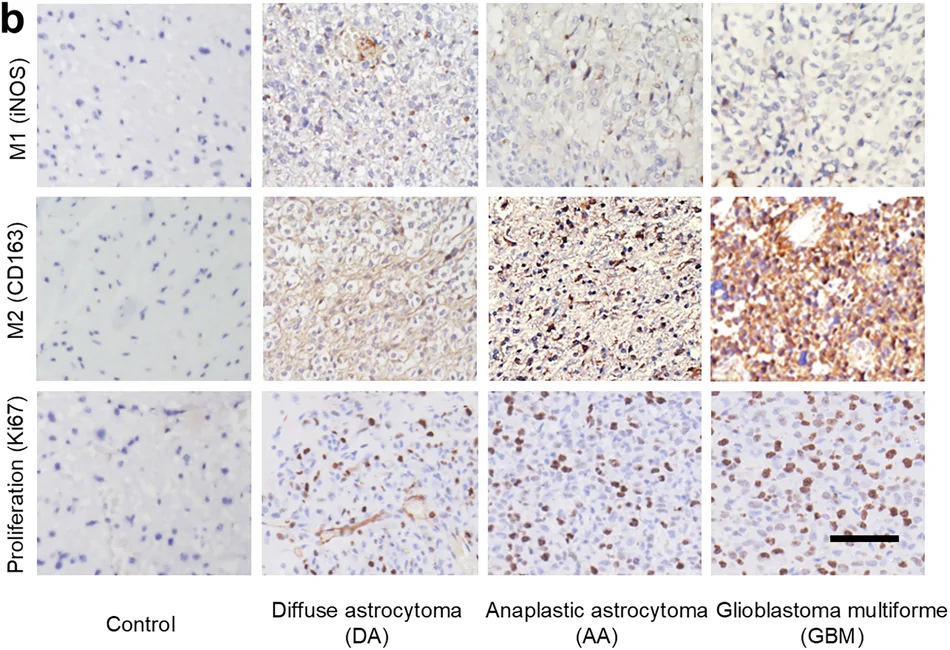

Within Supplementary Fig. S13, the HE staining images of the heart (M1EVs group) and brain (CCA-M1EVs group) were incorrectly pasted. This oversight occurred during data sorting and figure assembly. The corrected figures are presented below.

Incorrect Fig. S13:

**Figure:**
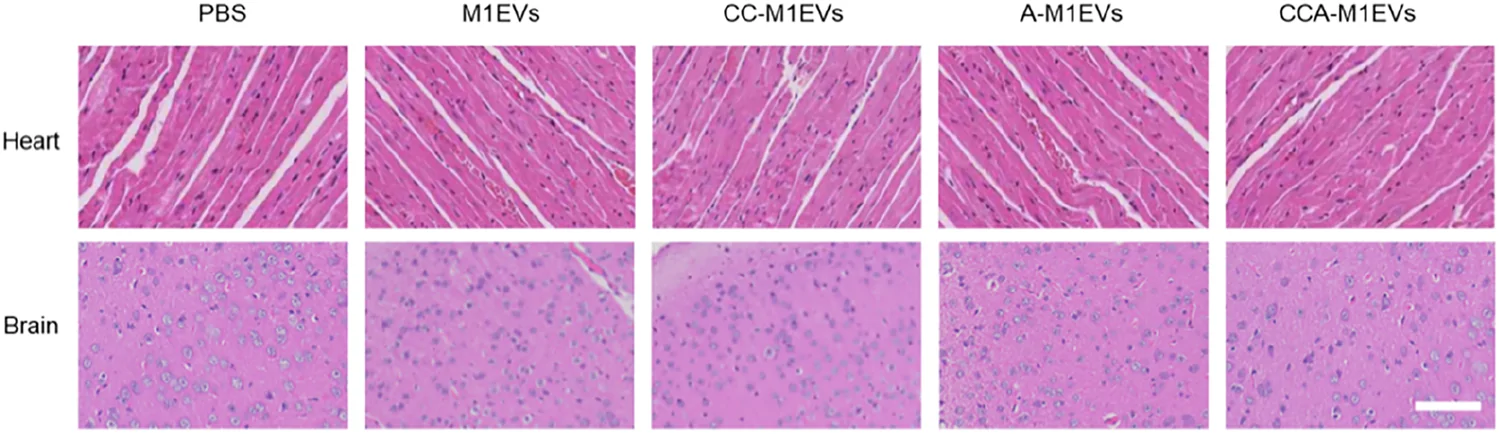

Correct Fig. S13:

**Figure:**
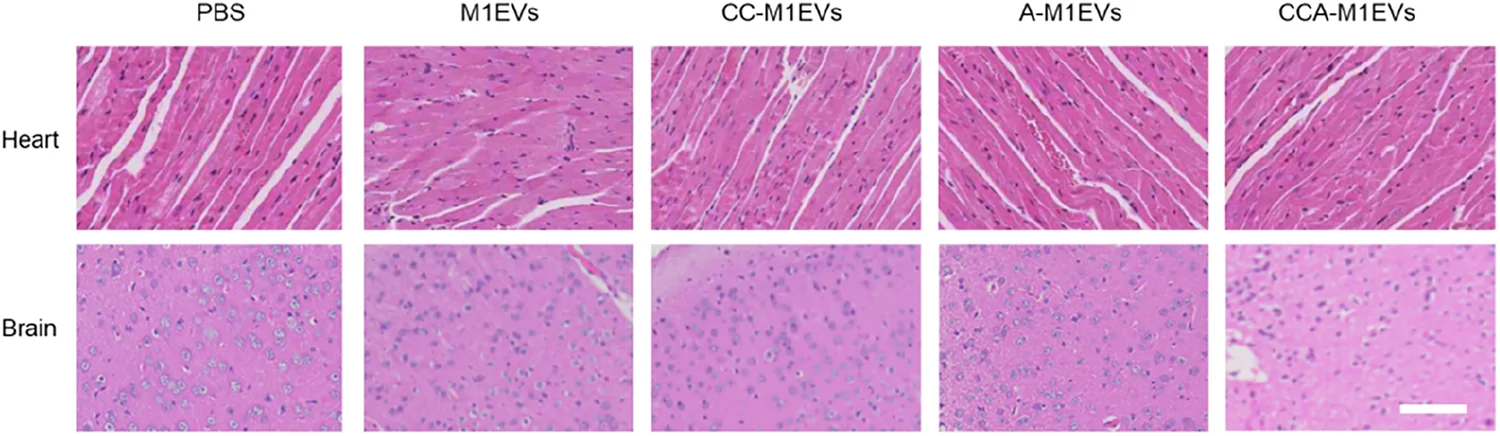

## Supplementary information

Supplementary Information
