# Supplementary material for: Correction: Exploration and functionalization of M1-macrophage extracellular vesicles for effective accumulation in glioblastoma and strong synergistic therapeutic effects
Source: Signal Transduct Target Ther. 2026 Sep 5;11:366. doi: 10.1038/s41392-026-02976-y (PMC13546281; doi:10.1038/s41392-026-02976-y)
Supplement: Supplementary file 1 [file 41392_2026_2976_MOESM1_ESM.pdf]

Supplementary Materials for

Exploration and functionalization of M1-macrophage extracellular vesicles for effective accumulation in glioblastoma and strong synergistic therapeutic effects

Xiaojun Wang[1,2,3], Hui Ding[4], Zongyang Li[1], Yaonan Peng[5], Hui Tan[1], Changlong Wang[2,6], Guodong Huang[1], Weiping Li[1*], Guanghui Ma[2,6*], Wei Wei[2,6*]

Correspondence to: Wei Wei: weiwei@ipe.ac.cn; Guanghui Ma: ghma@ipe.ac.cn;

Weiping Li: wpli@szu.edu.cn

This PDF file includes:

Figures S1 to S17
Tables S1 to S2

Supplementary Figures

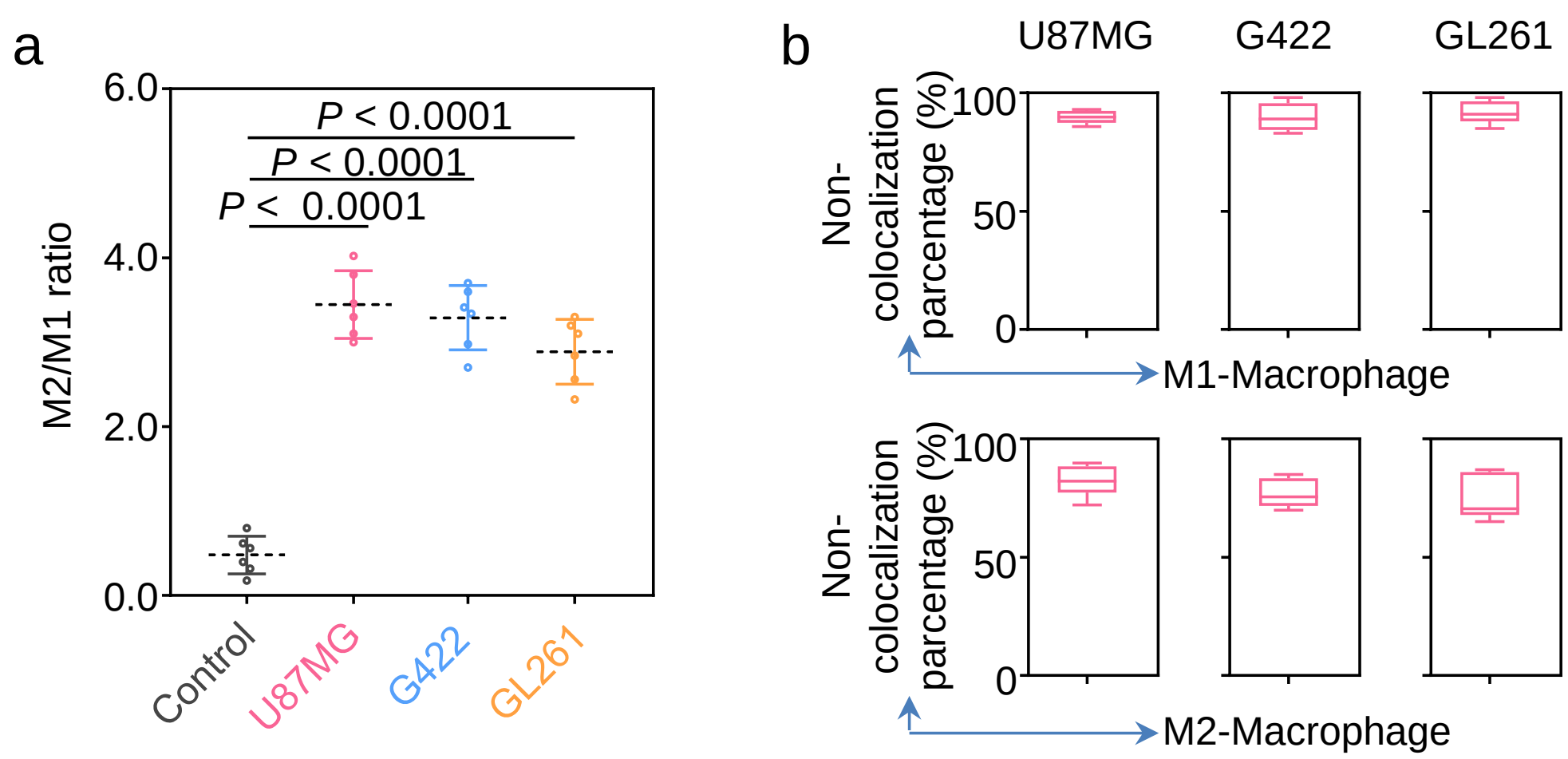

Fig. S1 Quantitative analysis of M2/M1 ratio in Fig. 1h and non-colocalization percentage of microglia and macrophage in Fig. 1i. a. Quantitative analysis of M2/M1 ratio of mouse glioma tissue in Fig. 1h (n=6). **b.** Quantification analysis of non-colocalization percentage of microglia and M1(M2)-like macrophage immunostaining of mouse glioma tissue (n=3). For **b**, data are presented as the mean ± S.D. Statistical significance between multiple groups was calculated using one-way ANOVA with a Kruskal-Wallis test.

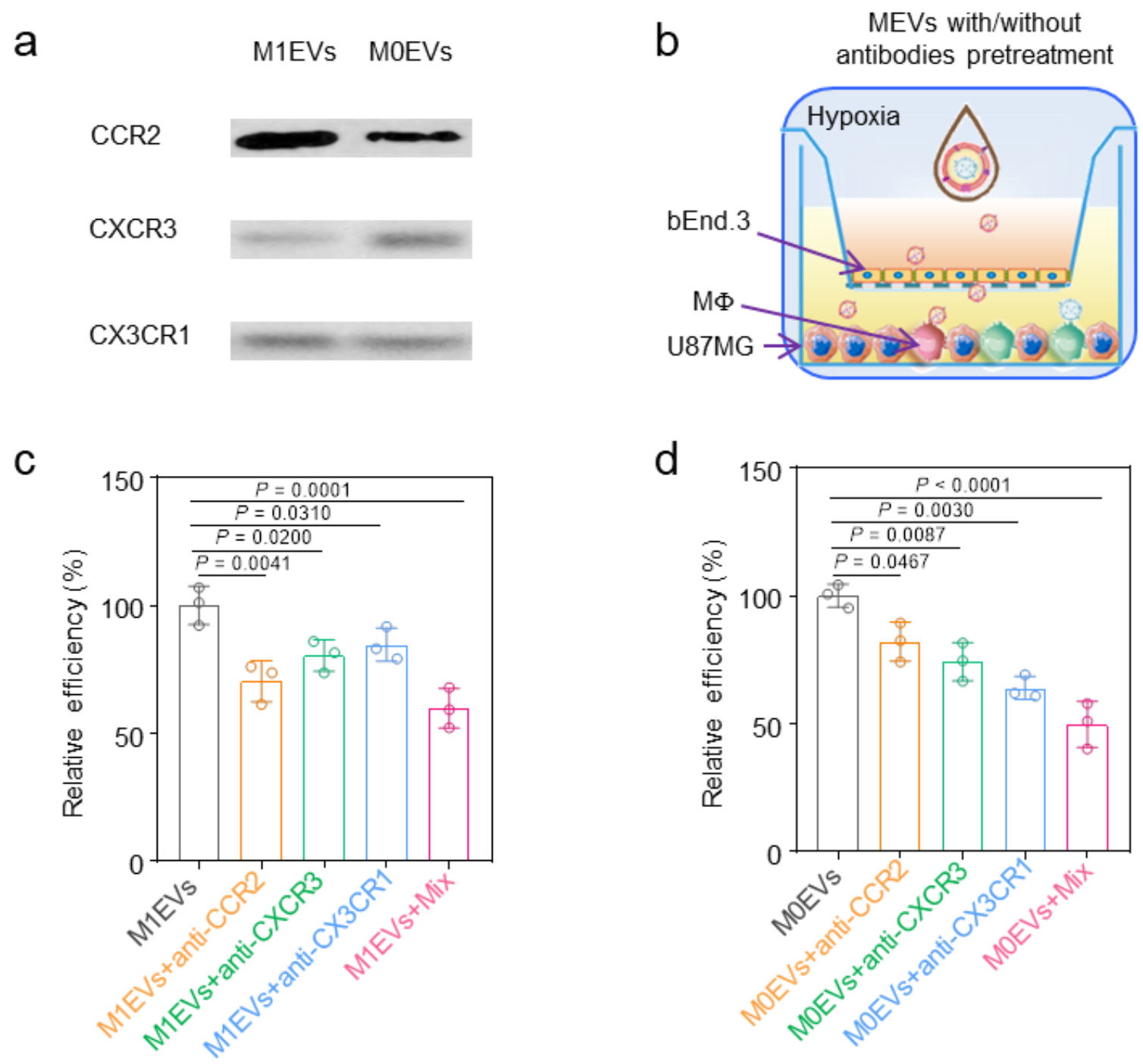

Fig. S2 *In vitro* evaluation of chemotactic properties of MEVs. a. Expression of chemokine receptors on MEV membranes by Western Blotting. **b.** Illustration of Transwell™ model for evaluation chemotactic properties of MEVs. **c.** Accumulative penetration efficiency of M1EVs, M1EVs+anti-CCR2, M1EVs+anti-CXCR3, M1EVs+anti-CX3CR1, and M1EVs+Mix (combination of anti-CCR2, anti-CXCR3, and anti-CX3CR1) in the lower chamber at 8 h (n=3). **d**. Accumulative penetration efficiency of M0EVs, M0EVs+anti-CCR2, M0EVs+anti-CXCR3, M0EVs+anti-CX3CR1, and M0EVs+Mix (combination of anti-CCR2, anti-CXCR3, and anti-CX3CR1) in the lower chamber at 8 h (n=3). After pretreatment with chemokine receptor antibodies, MEVs' penetration efficiencies were inhibited, which indicated MEVs' accumulation at glioma sites highly associated to the interaction of chemokine receptors on the MEVs and the corresponding chemokines produced by glioma. For **c** and **d**, data are presented as the mean ± S.D. Statistical significance was calculated by one-way ANOVA with a Kruskal-Wallis test.

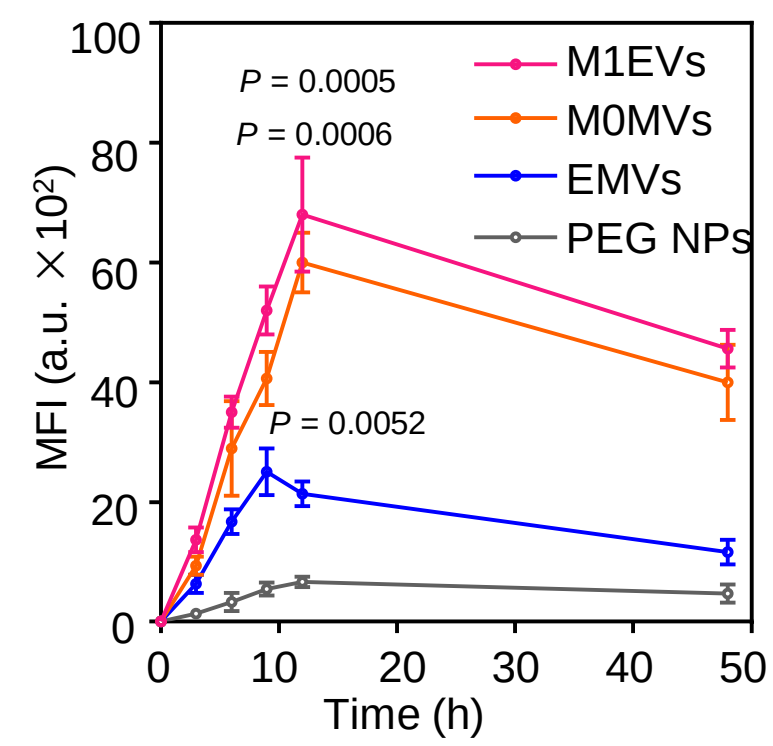

Fig. S3 Quantitative analysis of mean fluorescence intensity (MFI) in brain tissues of Fig. 2b. All data are presented as the mean ± S.D. (n=3). Statistical significance between multiple groups was calculated using one-way ANOVA with a Kruskal-Wallis test (12 h).

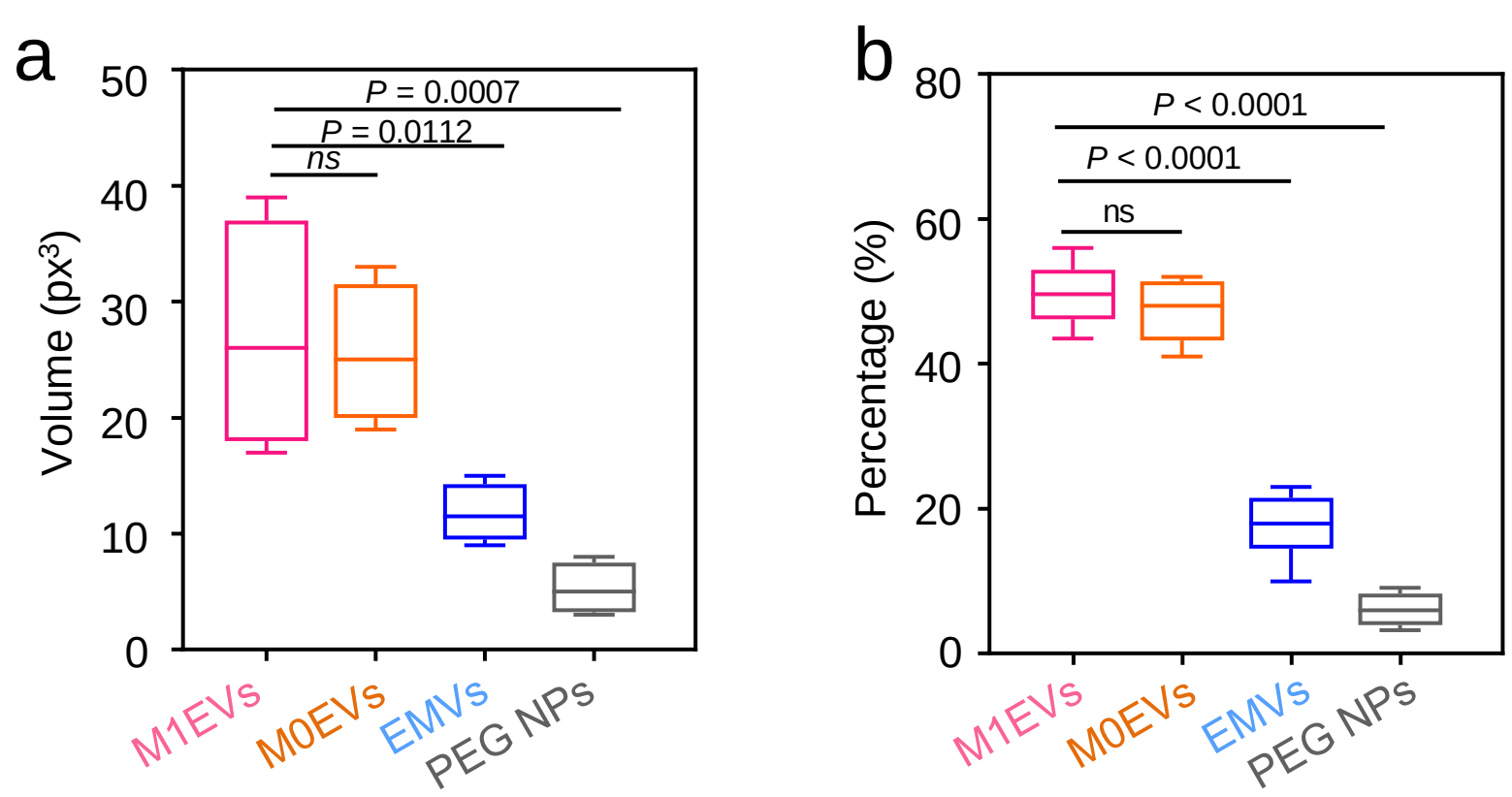

Fig. S4 Quantitative analysis of the mean diffusion volume in Fig. 2e (left) and particle distributions in Fig. 2e (right). a. Quantitative analysis of the mean diffusion volume of two-photon images in Fig. 2e. **b.** Quantitative analysis of particle distributions in Fig. 2e. Data are presented as the mean ± S.D. (n=3), and the center line shows the mean value. Statistical significance between multiple groups was calculated using one-way ANOVA with a Kruskal-Wallis test.

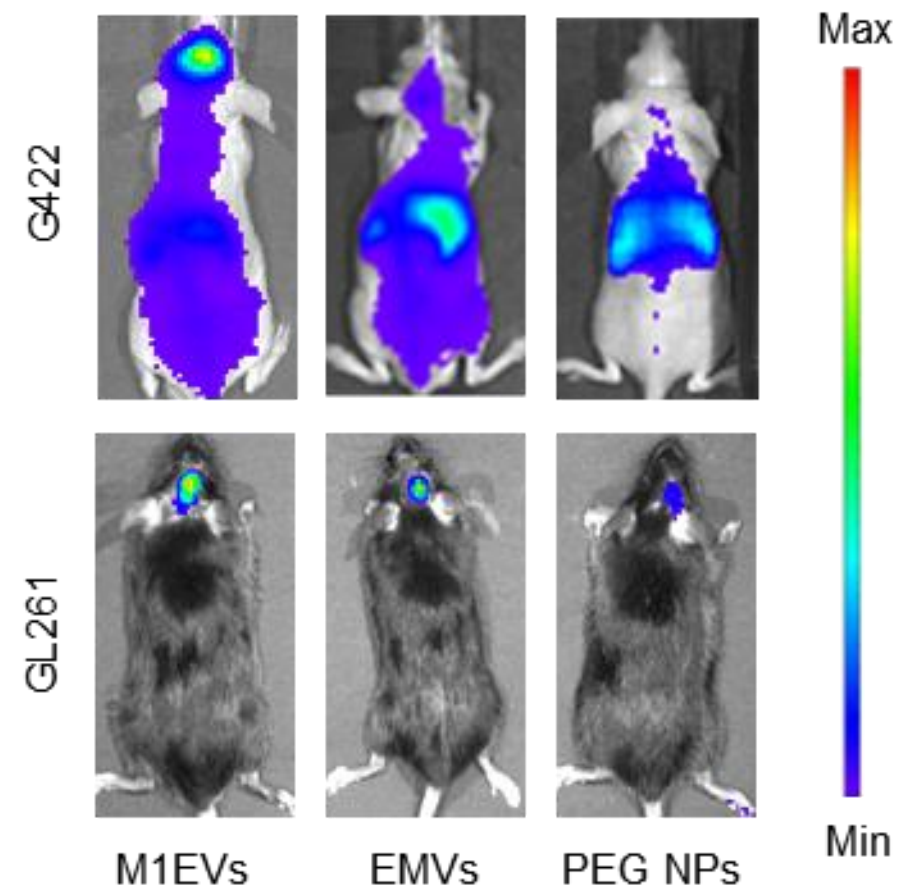

Fig. S5 Representative fluorescence images of G422/GL261-bearing mice after intravenous (*i.v.*) injection with M1EVs (mouse source), EMVs, and PEG NPs (all labeled with DiR) at 48 h. The tumor accumulation of M1EVs was more pronounced than that of the EMVs and PEG NPs.

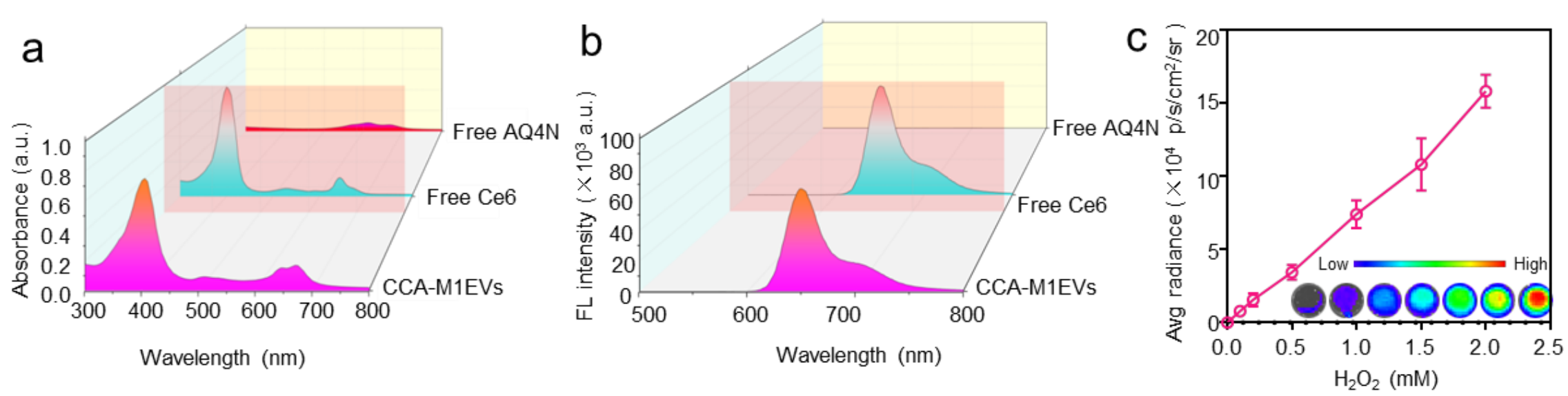

Fig. S6 The absorption, fluorescence spectra and chemluminescence of CCA-M1EVs. a. UV-vis spectrum of CCA-M1EVs, free Ce6, and free AQ4N. **b.** Fluorescence (FL) spectra of CCA-M1EVs, free Ce6, and free AQ4N. **c.** Chemiluminescence of CCA-M1EVs upon incubation with various concentrations of H_2O_2 (n=3). The successful encapsulation of AQ4N and Ce6 into CCA-M1EVs was confirmed by their characteristic absorbance peaks at 610 and 404 nm, respectively, on their UV-vis-NIR absorbance spectrum and fluorescence spectrum. These data, combined with chemiluminescence of CCA-M1EVs confirmed the proper assembly and functionalization of the CCA-M1EVs. The experiments in **a** and **b** were performed three times with similar results. For **c**, data are presented as the mean ± S.D.

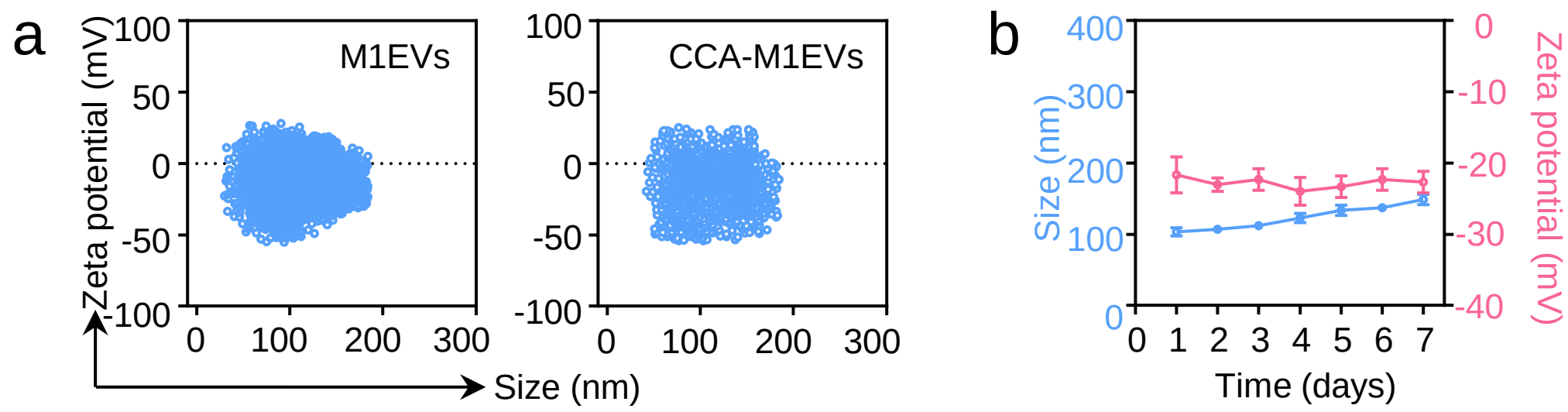

Fig. S7 Particle size, zeta potential, and stability of M1EVs and CCA-M1EVs. a. The size and zeta potentials of M1EVs and CCA-M1EVs. **b.** Stability investigation of CCA-M1EVs over a period of 7 days incubation in PBS buffer based on size and zeta potential (n=3). NTA analysis found that the size distribution and zeta potential of M1EVs before and after drug loading changed negligibly. To test the stability of drug-loaded EVs over time, we measured zeta potentials and average sizes (*via* NTA) of CCA-M1EVs in PBS once each day for 7 days, which revealed the CCA-M1EVs to be highly stable for intravenous injection. For **b**, data are presented as the mean ± S.D.

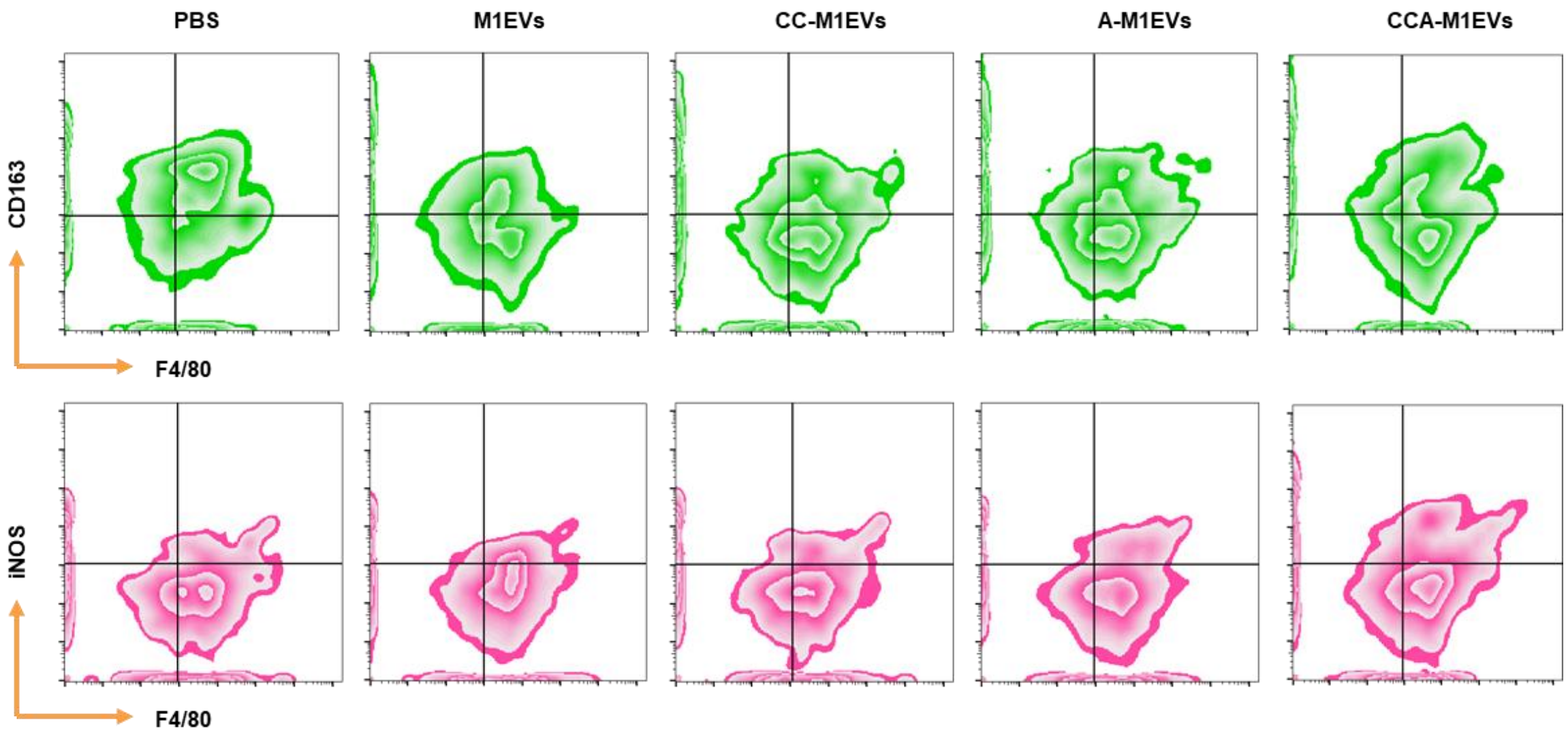

Fig. S8 ***In vitro*** **M2-to-M1 polarization of different formulations.** Representative flow cytometry analysis images of M2-like & M1-like macrophages (M2-$CD163^{+}iNOS^{-}$, M1-$CD163^{-}iNOS^{+}$, gated from $CD45^{+}CD11b^{+}$ cells) in the lower chamber after incubation with different EVs designs.

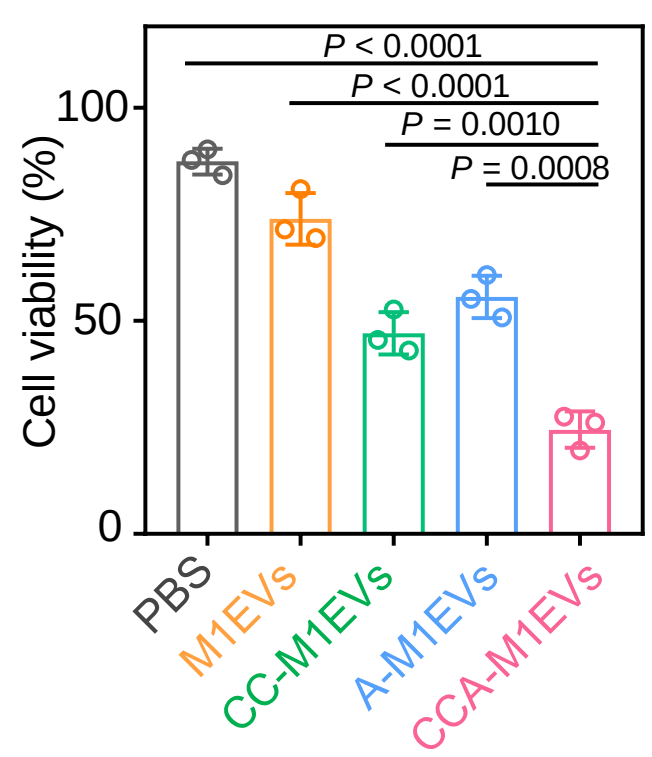

Fig. S9 ***In vitro*** **evaluation of anti-tumor effects.** CCK-8 analysis was utilized to evaluate the cytotoxicity of U87MG cells, and the treatments included PBS, M1EVs, CC-M1EVs, A-M1EVs, and CCA-M1EVs (n=3). The CCA-M1EVs group showed the most effective tumor cell viability inhibition. Data are presented as the mean ± S.D. Statistical significance was calculated by one-way ANOVA with a Kruskal-Wallis test.

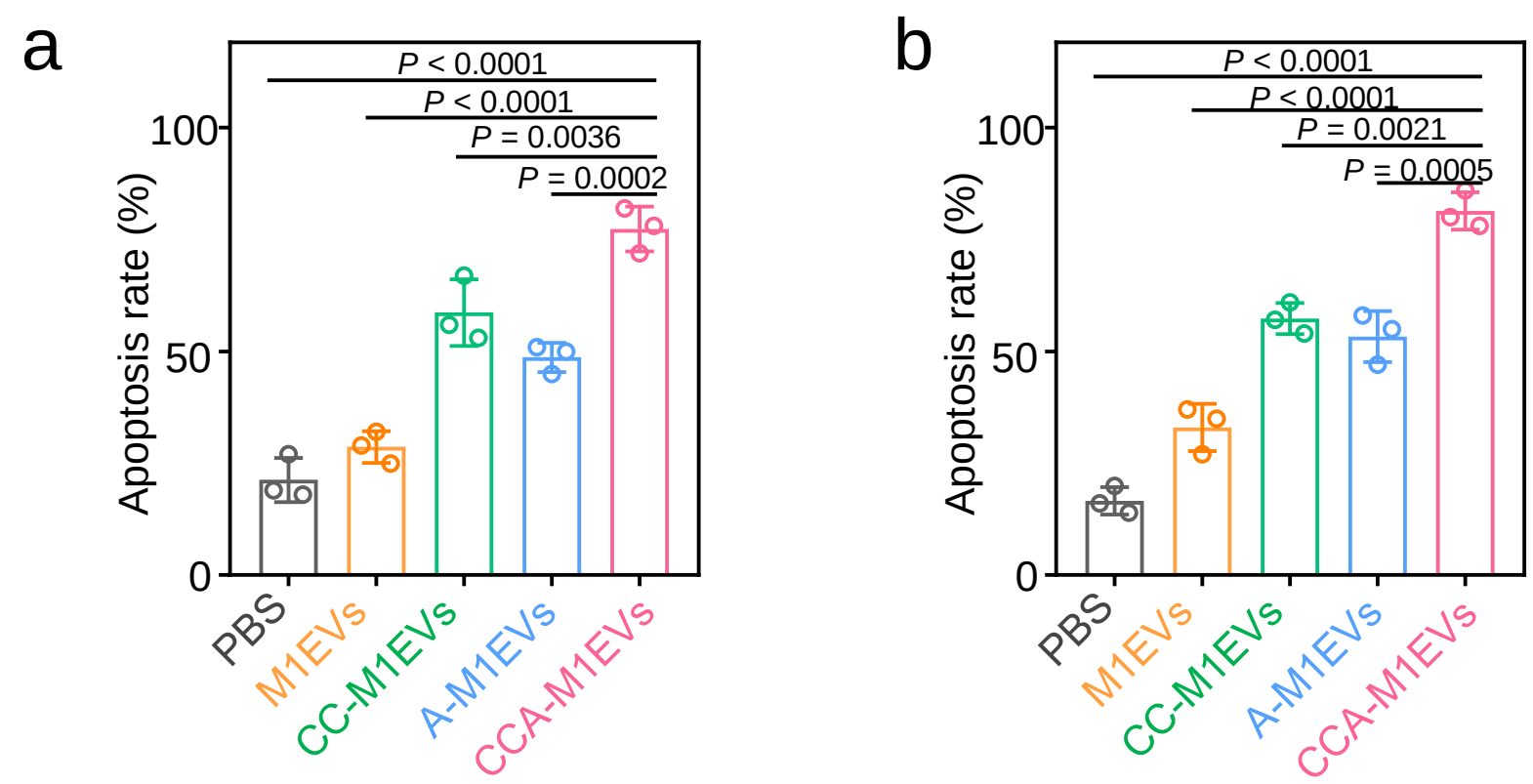

Fig. S10 Mouse glioma cells (G422/GL261) killing by different formulations. Flow cytometry analysis to assess the cell-death-inducing effect of different formulations (mouse source) on G422 cells (**a**) and GL261 cells (**b**) in the lower chamber (Annexin V and PI in the dead cell apoptosis kit) (n=3). Compared with other counterparts, CCA-M1EVs were observed with potent killing efficiency for G422 and GL261 cells. Statistical significance between multiple groups was calculated using one-way ANOVA with a Kruskal-Wallis test.

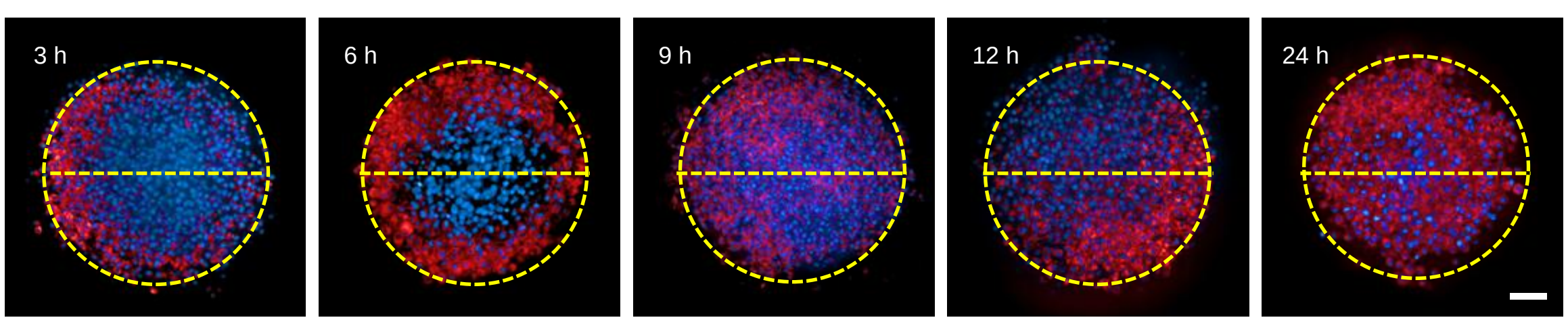

Fig. S11 Representative CLSM images of M1EVs penetration in MCTSs at different time points. CLSM imaging of the M1EVs into MCTSs over a 24 h incubation showed time-dependent increases in the extent of M1EVs penetration into spheroids. The experiment was performed three times with similar results. All images have the same scale of 50 μm.

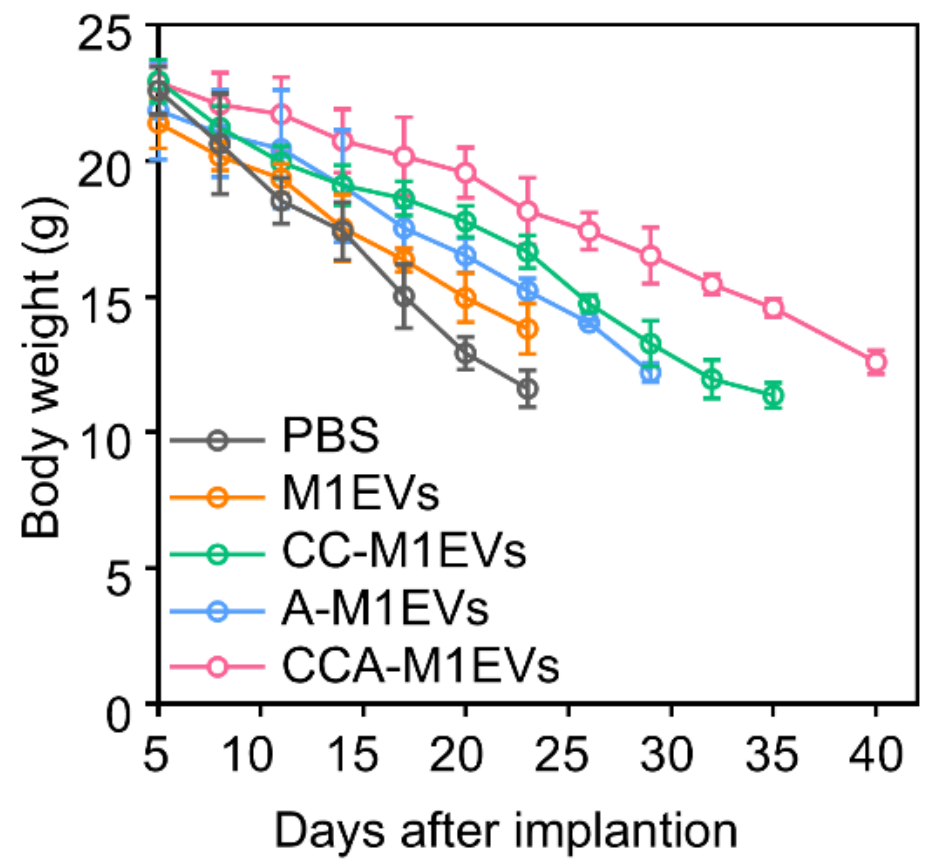

Fig. S12 Body weight changes during treatment stage. CCA-M1EVs showed a delayed body-weight loss relative to the other groups, indicating that this treatment effectively inhibited glioma growth without causing systemic adverse effect. All data are presented as the mean ± S.D. (n=8).

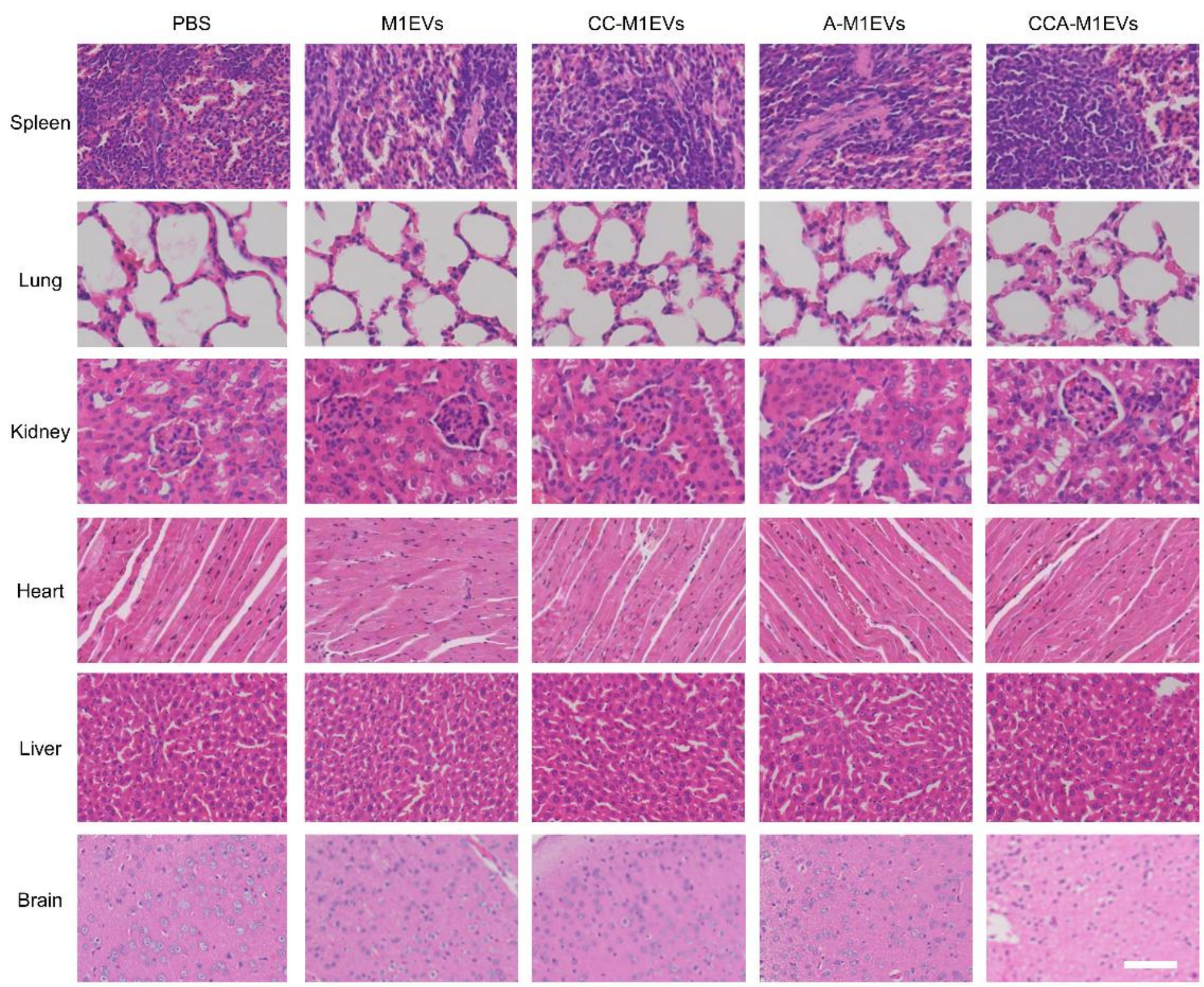

Fig. S13 *In vivo* toxicity evaluation of different formulations. These results exhibited no detectable pathological change, which confirmed the safe use of our nanoplatform. The experiment was performed three times with similar results. All images have the same scale of 50 μm.

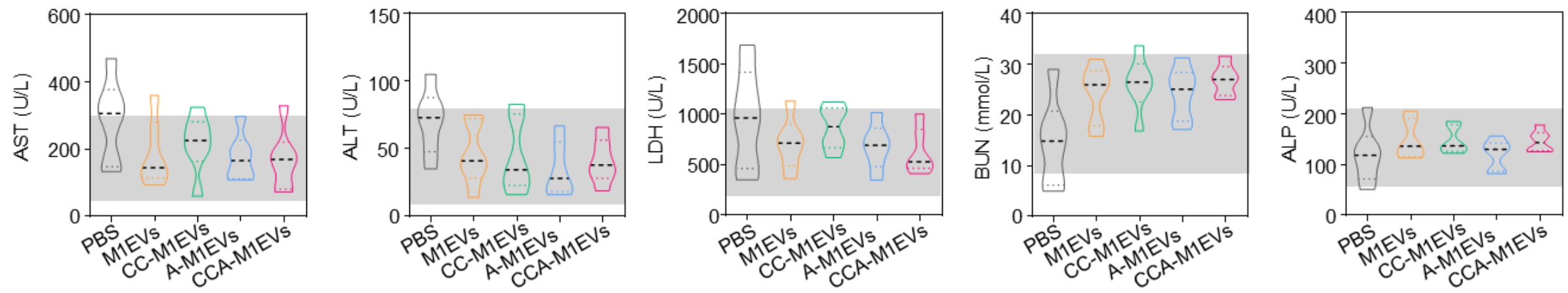

Fig. S14 Hematological analysis of U87MG-bearing mice after different treatments. Compared with the PBS group, the aminotransferase (AST), aspartate alanine aminotransferase (ALT), lactate dehydrogenase (LDH), blood urea nitrogen (BUN) and alkaline phosphatase (ALP) levels of the other groups were all within normal ranges. All data are presented as the mean ± S.D. (n=6). The floating bars extend from the minimum to maximum, and the center line shows the mean value.

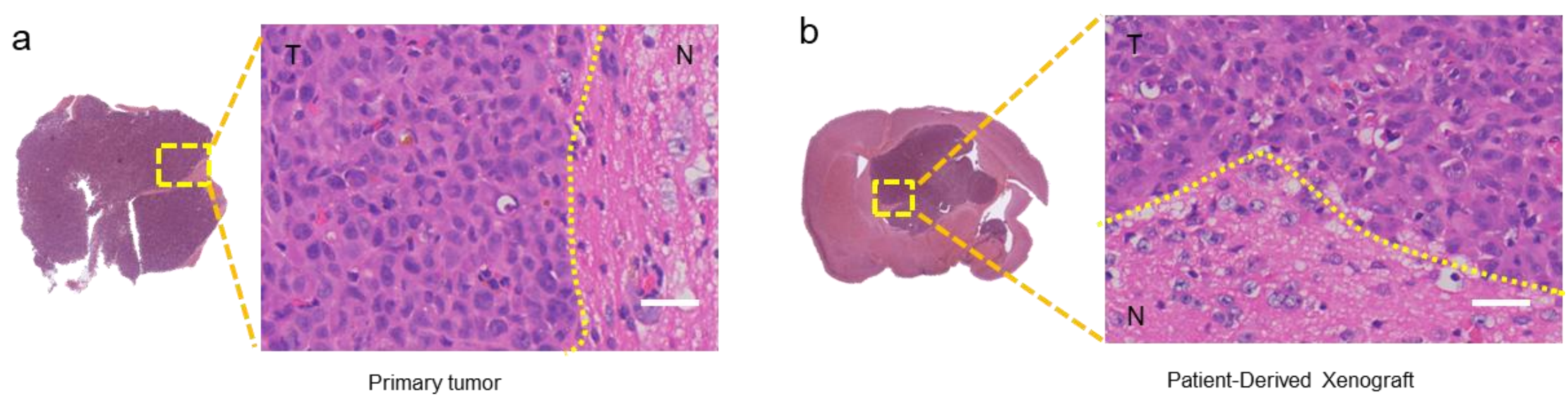

Fig. S15 H&E-stained slice images of patient-derived tissue (a) and PDX model (b). H&E staining showed that the PDX had the characteristics of the primary tumor. The experiment was performed three times with similar results. Scale bars: 100 μm. The yellow line indicate tumor boundary.

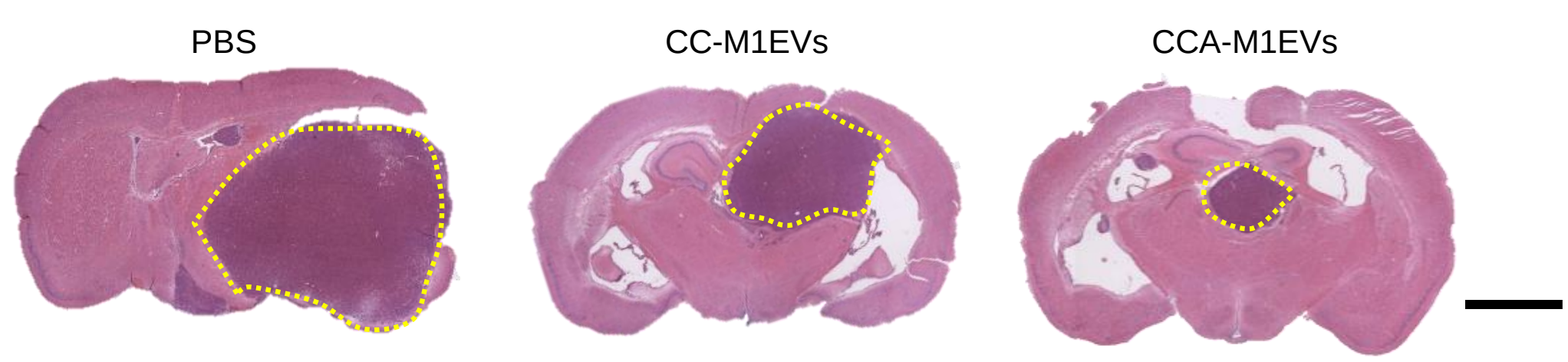

Fig. S16 Images of H&E staining of various groups in PDX tumor sections after 20 days. Compared with the PBS group, mice treated with CCA-M1EVs showed the greatest tumor inhibiton with the smallest tumor volume. The experiment was performed three times with similar results. Scale bar: 2 mm. The yellow line indicate tumor tissues.

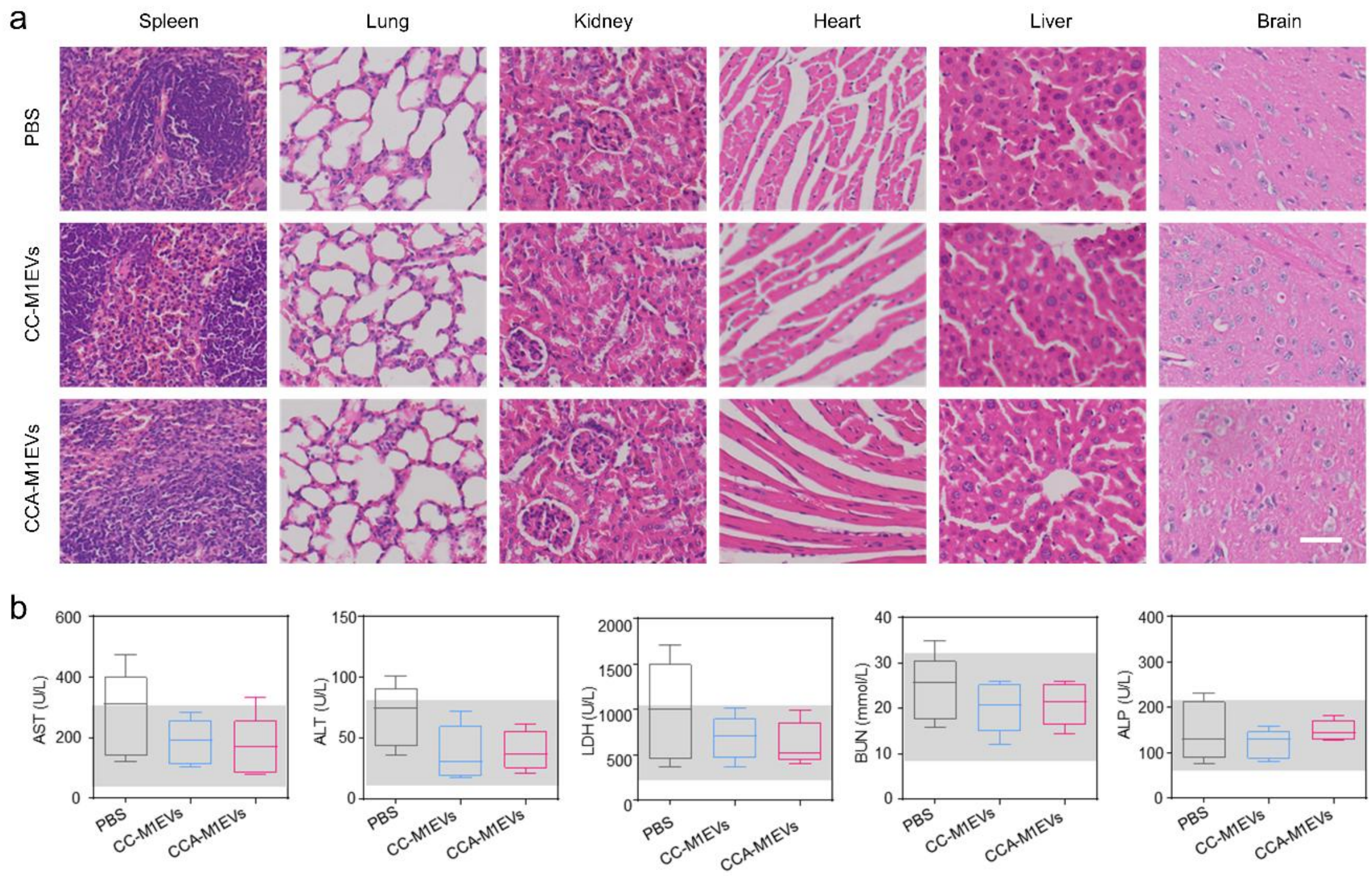

Fig. S17 *In vivo* toxicity evaluation of different treatments in PDX model. a. H&E-stained slice images of major organs from different groups (human PBMC source) after 20 days. These H&E results exhibited no detectable pathological change. **b.** Hematological analysis of different formulations treated PDX mice after 20 days (n=5). Compared with the PBS group, the AST, ALT, LDH, BUN and ALP levels of the other groups were all within normal ranges. The experiment in **a** was performed three times with similar results. All images have the same scale of 50 μm. For **b**, data are presented as the mean ± S.D., and the center line shows the mean value.

Supplementary Table

Table S1 Demographic and tumor characteristics of glioma patients

Case ID	Age at Diagnosis	Gender	Clinical Diagnosis	Pathological Diagnosis	WHO Grade	Ki67	M2/M1 (Ratio)
DA1	23	Male	Diffuse right frontal astrocytoma	Diffuse astrocytoma	WHO II	5%+	1.63
DA2	30	Male	Bilateral ventricular glioma	Diffuse astrocytoma	WHO II	3%+	1.28
DA3	39	Female	Diffuse astrocytoma of right frontotemporal insula	Diffuse astrocytoma	WHO II	2%+	1.51
DA4	33	Female	Left (side) frontal glioma	Diffuse astrocytoma	WHO II	8%+	2.26
DA5	31	Male	Diffuse astrocytoma of left frontotemporal insula	Diffuse astrocytoma	WHO II	2%+	1.18
DA6	41	Male	Left temporal lobe insular astrocytoma	Diffuse astrocytoma	WHO II	3%+	1.29
DA7	31	Female	Right insular lobe astrocytoma	Diffuse astrocytoma	WHO II	3%+	1.36
DA8	30	Female	Diffuse astrocytoma of right frontal lobe	Diffuse astrocytoma	WHO II	8%+	1.63
DA9	32	Female	Diffuse astrocytoma of right frontal lobe	Diffuse astrocytoma	WHO II	5%+	1.58
DA10	28	Female	Polymorphic yellow astrocytoma of the left temporal lobe	Diffuse astrocytoma	WHO II	3%+	1.74
DA11	30	Female	Right frontotemporal astrocytoma	Diffuse astrocytoma	WHO II	5%+	1.91
DA12	26	Female	Right frontal lobe glioma	Diffuse astrocytoma	WHO II	5%+	1.99
DA13	38	Female	Diffuse astrocytoma of right frontotemporal insula	Diffuse astrocytoma	WHO II	5%+	1.49
DA14	20	Female	Right temporal lobe glioma	Diffuse astrocytoma	WHO II	2%+	1.73
DA15	37	Male	Right frontal parietal glioma	Diffuse astrocytoma	WHO II	3%+	1.49
DA16	26	Male	Left frontal glioma	Diffuse astrocytoma	WHO II	8%+	1.62
DA17	18	Male	Right occipital lobe glioma	Diffuse astrocytoma	WHO II	5%+	1.50
DA18	26	Female	Central neurocytoma of left ventricle	Diffuse astrocytoma	WHO II	20%+	2.99
DA19	64	Female	Left frontal lobe glioma	Diffuse astrocytoma	WHO II	10%+	2.13
DA20	44	Male	Right frontal and temporal astrocytoma	Diffuse astrocytoma	WHO II	2%+	1.48
DA21	34	Male	Left frontal lobe oligodendroastrocytoma	Diffuse astrocytoma	WHO II	5%+	2.74
DA22	26	Male	Diffuse astrocytoma of right frontotemporal insula	Diffuse astrocytoma	WHO II	5%+	1.93
AA1	58	Female	Right frontal anaplastic oligodendroglioma	Anaplastic astrocytoma	WHO III	8%+	1.31
AA2	32	Male	Right frontal parietal insula anaplastic astrocytoma	Anaplastic astrocytoma	WHO III	20%+	2.82
AA3	26	Female	Frontal anaplastic astrocytoma	Anaplastic astrocytoma	WHO III	15%+	3.07
AA4	46	Male	Brainstem anaplastic astrocytoma	Anaplastic astrocytoma	WHO III	10%+	2.13
AA5	37	Female	Left frontal diffuse astrocytoma	Anaplastic astrocytoma	WHO III	15%+	2.25
AA6	34	Female	Recurrent glioma of right frontal parietal lobe	Anaplastic astrocytoma	WHO III	20%+	2.77
AA7	65	Female	Left frontal glioma	Anaplastic astrocytoma	WHO III	5%+	1.81
AA8	72	Female	Anaplastic astrocytoma	Anaplastic astrocytoma	WHO III	20%+	3.44
AA9	48	Female	Left parietal oligodendroglioma	Anaplastic astrocytoma	WHO III	10%+	2.60
AA10	66	Female	Right frontotemporal insular degeneration astrocytoma	Anaplastic astrocytoma	WHO III	30%+	2.57
AA11	44	Female	Right frontal anaplastic astrocytoma	Anaplastic astrocytoma	WHO III	10%+	1.84
AA12	55	Female	Bilateral frontal lobe oligodendroglioma partial anaplasia	Anaplastic astrocytoma	WHO III	8%+	2.02
AA13	47	Male	Right frontal lobe tumor	Anaplastic astrocytoma	WHO III	10%+	2.19
AA14	24	Female	Left frontal glioma	Anaplastic astrocytoma	WHO III	15%+	2.42
AA15	45	Female	Anaplastic astrocytoma	Anaplastic astrocytoma	WHO III	15%+	2.48
AA16	28	Male	Left frontal anaplastic oligodendroglial astrocytoma	Anaplastic astrocytoma	WHO III	10%+	2.54
AA17	49	Female	Anaplastic astrocytoma	Anaplastic astrocytoma	WHO III	10%+	2.07
AA18	32	Female	Left frontal and left frontal-temporal glioma	Anaplastic astrocytoma	WHO III	5%+	1.38
AA19	52	Male	Right parieto-occipital lobe glioma	Anaplastic astrocytoma	WHO III	30%+	2.99
AA20	54	Male	Left frontotemporal insular glioma	Anaplastic astrocytoma	WHO III	30%+	2.47
GBM1	56	Male	Glioblastoma multiforme of bilateral frontal lobe and corpus callosum	Glioblastoma multiforme	WHO IV	25%+	2.53
GBM2	70	Male	Right parietal glioblastoma	Glioblastoma multiforme	WHO IV	40%+	3.58
GBM3	60	Male	Left frontal glioblastoma	Glioblastoma multiforme	WHO IV	30%+	2.84
GBM4	43	Male	Right temporal occipital lobe glioblastoma	Glioblastoma multiforme	WHO IV	60%+	3.34
GBM5	44	Male	Glioblastoma multiforme	Glioblastoma multiforme	WHO IV	40%+	4.11
GBM6	51	Male	High-grade astrocytoma of the left temporal parietal corpus callosum	Glioblastoma multiforme	WHO IV	30%+	3.08
GBM7	66	Male	Bilateral frontal and corpus callosum: glioblastoma	Glioblastoma multiforme	WHO IV	12%+	1.78
GBM8	34	Male	Left (side) frontal lobe glioblastoma	Glioblastoma multiforme	WHO IV	30%+	3.23
GBM9	55	Female	Right temporal glioblastoma	Glioblastoma multiforme	WHO IV	40%+	3.46
GBM10	47	Male	Glioblastoma multiforme	Glioblastoma multiforme	WHO IV	40%+	2.36
GBM11	64	Male	Right temporal lobe glioblastoma	Glioblastoma multiforme	WHO IV	30%+	3.02
GBM12	57	Female	Glioblastoma of left frontotemporal basal ganglia	Glioblastoma multiforme	WHO IV	30%+	2.65
GBM13	33	Male	Left frontotemporal insula glioma	Glioblastoma multiforme	WHO IV	50%+	2.77
GBM14	28	Female	Left thalamic glioblastoma	Glioblastoma multiforme	WHO IV	30%+	2.82
GBM15	51	Male	Right frontal lobe glioblastoma	Glioblastoma multiforme	WHO IV	30%+	2.88
GBM16	38	Female	Glioblastoma of left thalamus lateral ventricle	Glioblastoma multiforme	WHO IV	30%+	3.17
GBM17	60	Male	Right parietal glioblastoma	Glioblastoma multiforme	WHO IV	15%+	3.40
GBM18	60	Female	Right frontal lobe glioblastoma	Glioblastoma multiforme	WHO IV	40%+	3.29
GBM19	38	Male	Right frontal lobe glioblastoma	Glioblastoma multiforme	WHO IV	30%+	2.72
GBM20	59	Male	Right temporal lobe glioblastoma	Glioblastoma multiforme	WHO IV	20%+	2.71
GBM21	32	Male	Left temporal parietal glioma	Glioblastoma multiforme	WHO IV	40%+	3.57
GBM22	43	Male	Left frontal lobe glioblastoma	Glioblastoma multiforme	WHO IV	35%+	3.28

Table S2 Patient characteristics of the PDX model

Case ID	Age at Diagnosis	Gender	Clinical Diagnosis	Pathological Diagnosis	WHO Grade	Ki67	M2/M1 (ratio)
GBM23	59	Female	Right frontal lobe glioblastoma	High grade glioma	III-IV	46%+	3.78
